# Characterisation of Staphylococci species from neonatal blood cultures in low- and middle-income countries

**DOI:** 10.1186/s12879-022-07541-w

**Published:** 2022-07-01

**Authors:** Kirsty Sands, Maria J. Carvalho, Owen B. Spiller, Edward A. R. Portal, Kathryn Thomson, William John Watkins, Jordan Mathias, Calie Dyer, Chinenye Akpulu, Robert Andrews, Ana Ferreira, Thomas Hender, Rebecca Milton, Maria Nieto, Rabaab Zahra, Haider Shirazi, Adil Muhammad, Shermeen Akif, Muhammad Hilal Jan, Kenneth Iregbu, Fatima Modibbo, Stella Uwaezuoke, Grace J. Chan, Delayehu Bekele, Semaria Solomon, Sulagna Basu, Ranjan Kumar Nandy, Sharmi Naha, Jean-Baptiste Mazarati, Aniceth Rucogoza, Lucie Gaju, Shaheen Mehtar, Andre N. H. Bulabula, Andrew Whitelaw, BARNARDS Group, Timothy R. Walsh

**Affiliations:** 1 Department of Medical Microbiology, Division of Infection and Immunity, School of Medicine, Cardiff University, Cardiff, UK; 2 Department of Zoology, Ineos Oxford Institute of Antimicrobial Research, Oxford, UK; 3 Institute of Biomedical Sciences, Department of Medical Sciences, University of Aveiro, Aveiro, Portugal; 4 Centre for Trials Research, Cardiff University, Cardiff, UK; 5 National Hospital Abuja, Abuja, Nigeria; 6 Department of Microbiology, Quaid-I-Azam University, Islamabad, Pakistan; 7 Pakistan Institute of Medical Sciences, Islamabad, Pakistan; 8 Murtala Muhammad Specialist Hospital, Kano, Nigeria; 9 Federal Medical Centre Jabi, Abuja, Nigeria; 10 Division of Medical Critical Care, Boston Children’s Hospital, Boston, MA USA; 11 Department of Epidemiology, Harvard T.H Chan School of Public Health, Boston, MA USA; 12 Department of Obstetrics and Gynecology, St Paul’s Hospital Millennium Medical College, Addis Ababa, Ethiopia; 13 Department of Microbiology, Immunology and Parasitology, St Paul’s Hospital Millennium Medical College, Addis Ababa, Ethiopia; 14 Division of Bacteriology, ICMR-National Institute of Cholera and Enteric Diseases Beliaghata, Kolkata, India; 15 Rwanda Biomedical Centre, The National Reference Laboratory, Kigali, Rwanda; 16 Unit of IPC, Stellenbosch University, Cape Town, South Africa; 17 Infection Control Africa Network, Cape Town, South Africa; 18 Department of Global Health, Stellenbosch University, Cape Town, South Africa; 19 Division of Medical Microbiology, Stellenbosch University, Cape Town, South Africa; 20 National Health Laboratory Service, Tygerberg Hospital, Cape Town, South Africa

**Keywords:** LMIC, Staphylococci, Neonatal sepsis, Genomics, *Mammaliicocci*, Mortality, Early onset, Late onset, Epidemiology

## Abstract

**Background:**

In low- and middle-income countries (LMIC) *Staphylococcus aureus* is regarded as one of the leading bacterial causes of neonatal sepsis, however there is limited knowledge on the species diversity and antimicrobial resistance caused by Gram-positive bacteria (GPB).

**Methods:**

We characterised GPB isolates from neonatal blood cultures from LMICs in Africa (Ethiopia, Nigeria, Rwanda, and South Africa) and South-Asia (Bangladesh and Pakistan) between 2015–2017. We determined minimum inhibitory concentrations and performed whole genome sequencing (WGS) on Staphylococci isolates recovered and clinical data collected related to the onset of sepsis and the outcome of the neonate up to 60 days of age.

**Results:**

From the isolates recovered from blood cultures, Staphylococci species were most frequently identified. Out of 100 *S. aureus* isolates sequenced, 18 different sequence types (ST) were found which unveiled two small epidemiological clusters caused by methicillin resistant *S. aureus* (MRSA) in Pakistan (ST8) and South Africa (ST5)*,* both with high mortality (n = 6/17). One-third of *S. aureus* was MRSA, with methicillin resistance also detected in *Staphylococcus epidermidis, Staphylococcus haemolyticus* and *Mammaliicoccus sciuri.* Through additional WGS analysis we report a cluster of *M. sciuri* in Pakistan identified between July-November 2017.

**Conclusions:**

In total we identified 14 different GPB bacterial species, however Staphylococci was dominant. These findings highlight the need of a prospective genomic epidemiology study to comprehensively assess the true burden of GPB neonatal sepsis focusing specifically on mechanisms of resistance and virulence across species and in relation to neonatal outcome.

**Supplementary Information:**

The online version contains supplementary material available at 10.1186/s12879-022-07541-w.

## Background

Infections are a major cause of neonatal mortality [1, 2] with over 200,000 deaths reported each year [3]. Globally there is a large variation in the incidence of neonatal sepsis, although the burden is greatest in low- and middle-income countries (LMICs) [4, 5]. Despite *Staphylococcus aureus* being frequently reported as one of the top three bacterial causes of neonatal sepsis in LMICs along with GNB including *Klebsiella pneumoniae* and *Escherichia coli* [2, 6–9], surveillance data particularly using whole genome sequencing (WGS) is scarce. Understanding the true burden of coagulase negative Staphylococci (CoNS) in neonatal sepsis is far more complex. Despite multiple studies over the past decade suggesting that *Staphylococcus epidermidis* and *Staphylococcus haemolyticus* are important causes of nosocomial and bloodstream infections in neonates [10–13], there is often little capacity to implement routine surveillance, and these species are in many cases still considered contaminants, as they commonly reside on skin.

Studies have shown that neonates born at a very low birth weight (VLBW, < 1,500 g) may have a greater risk of contracting CoNS [11, 14]. Over the past two decades the number of specialised neonatal units in LMICs has fortunately increased, further increasing survival of VLBW infants. However, the likelihood of neonates exposed to nosocomial acquired CoNS is therefore higher, due to increased hospital stay for VLBW infants. Microbial investigation of CoNS isolated from blood cultures in countries with limited resources is often problematic due to a lack of simple and cost-effective methods of identification [9, 15]. Even if laboratories are equipped with manual or automated biochemical tests, these are laborious leading to subjective and inaccurate results; the tests add to the laboratory costs, and this may be difficult to justify given the often uncertain significance of the isolates. Although studies have reported cases of *Staphylococcus epidermidis* and *Staphylococcus haemolyticus* neonatal sepsis [9, 12, 13] and a PubMed search of “Staphylococci neonatal sepsis” produced over 300 hits (the majority of studies conducted in high-income countries) there is still a lack of data revealing the genomic diversity and resistome and virulome of staphylococcal species in LMICs.

BARNARDS is a network of 12 clinical sites distributed among four African (Ethiopia, Nigeria, Rwanda, South Africa) and three South-Asian (Bangladesh, India, Pakistan) countries aiming to assess the burden of antimicrobial resistance (AMR) in neonates. Herein we report data on sepsis caused by Gram-positive bacteria (GPB). We focus on the reporting of genomic diversity, genomic traits including antimicrobial resistance and virulence genes, and the phenotypic susceptibility profiles against 14 antibiotics for *S. aureus* isolates*.* Where possible, we performed genomics analysis on CoNS isolates to disclose AMR genotypes and whether mobile genetic elements were present. Finally, using WGS we report on an epidemiological cluster of neonatal sepsis caused by *Mammaliicoccus sciuri,* previously known as *Staphylococcus sciuri* in Islamabad, Pakistan between July-November 2017.

## Methods

### Study design

Ethical approval was obtained prior to the start of the study (Additional file 1: Table S1). We have used the term ‘neonate’ for all enrolments, this includes up to 60 days of age. Upon clinical signs of sepsis, a blood culture (using automated blood culture systems either BD BACTEC™ or BacT/ALERT® bioMérieux) was collected and processed using automated blood culture systems. Early onset sepsis (EOS) was defined as sepsis occurring < 72 h and late onset sepsis (LOS) > 72 h after birth. Neonates were followed up until 60 days. Clinical data collected included onset and outcome of sepsis. Due to high reporting of GPB sepsis and in particular *S. aureus* during a BARNARDS network meeting in 2017 (a program originally designed with an emphasis on carbapenem resistance in GNB neonatal sepsis) we retrospectively recovered GPB isolates for genomics characterisation in addition to GNB [16]. Unfortunately, at this point, 970/1266 GPB isolates, predominantly non-*S. aureus* were not stored or recoverable. Blood isolates were stored on charcoal swabs (Deltalab) for transport under UN3373 regulations to Cardiff University, UK.

### Antimicrobial susceptibility testing

In Cardiff, GPB were plated onto Columbia Blood Agar supplemented with 5% sterile sheep/horse blood and incubated aerobically at 37** °C** for 24–48 h. Isolates were identified using Microflex LT MALDI-TOF MS (Bruker Daltonik, GmbH, UK) with α-Cyano-4-hydroxycinnamic acid (HCCA) matrix (Sigma Aldrich) and stored in TS/72 beads (Technical Service Consultants, UK) at -80 °C. Minimum inhibitory concentrations (MICs) were determined by in-house agar dilution for 14 antibiotics (Additional file 1: Table S2) using *Staphylococcus aureus* ATCC 29,213, *Escherichia coli* ATCC 25,922 and *Pseudomonas aeruginosa* ATCC 2785 for quality control and interpreted according to the EUCAST v11 guidelines [17]. The MIC_50_ and MIC_90_ for each antibiotic was determined for *S. aureus.* Each bacterial isolate was tested once per antibiotic concentration to generate a single data point following validation using appropriate control strains listed above.

### Whole genome sequencing and bioinformatics analysis

#### Assembly and annotation

WGS, assembly and annotation were performed as previously described [16], and individual accession numbers are listed in Additional file 1: Table S3. On average, isolates were paired-end sequenced to > 40 × coverage using Nextera XT v2 on an Illumina Miseq generating 300 bp reads. A summary of the bioinformatics programs and parameters used in this study can be found in Additional file 1: Fig. S1. Genome coverage was assessed using Qualimap (v2.2.1) [18]. ABRicate (v0.9.7) [19] (> 98% coverage and identity) was used to detect antimicrobial resistance genes (ARG) [20] and virulence factors (VF) [21]. Species identification was confirmed using BLAST v2.7.1 [22]. In silico MLST was determined for *S. aureus, S. epidermidis* and *S. haemolyticus* using mlst (v2.17.6) [23]. Novel alleles and profiles were submitted using PubMLST [24]. *Staphylococcus aureus* specific staphylococcal protein A (spa) gene typing, IS*256* screening, and SCC*mec* cassette determination was performed using CGE [25] in combination with the following databases; *spa* typer (v1.0) [26], MGE (v1.0.2) [27] and SCCmecFinder (1.2) [28].

#### Comparative genomics

*S. aureus* (n = 251) from a European collection by Aanensen et al*.,* 2016 [29] were incorporated into a species wide analysis using Prokka (v1.14.0) [30], Roary (v3.12.0) [31] and FastTree (v2.1.11) [32]. For all core genome SNP analyses performed (depicted in Additional file 1: Fig. S1), a representative reference of each dominant ST was selected from the genomes submitted to NCBI marked as ‘complete’, for variant calling using Snippy (v4.0.5) [33], Gubbins (v2.3.4) [34] snp-sites (v2.5.1)[35] and Raxml-ng (v0.9.0) [36] and IQ-tree (v2.0) where specified [37]. SNP pairwise distances were generated using snp-dists (v0.6) [38]. Phylogenetic trees were mid-rooted and annotated using iTOL (v5.7) [39]. In total n = 2,327 *S. epidermidis* and n = 310 *S. haemolyticus* genomes were downloaded from NCBI on 28^th^ October 2020. Strains of the same ST (assessed using mlst v.2.17.6 [23]) of those found within this study were retained for comparative analysis. Metadata on whether these were reported as clinical, commensal, or other was recorded (Additional file 1: Table S4). SNP phylogeny workflows were performed as described above.

#### WGS analysis of *Mammaliicoccus sciuri*

An isolate belonging to the cluster was selected for complementary long read sequencing to produce a high-quality reference. gDNA was extracted and quantified as described [16], and SPRI beads (Mag-Bind TotalPure, Omega) were used to concentrate and purify at a 1:1 ratio with a final elution volume of 15µL to achieve an optimal range between 40-60 ng/µL. gDNA was quantified with the dsDNA BR assay kit using the qubit 4.0 fluorometer (Thermofisher). Genomic libraries were prepared using the Rapid Barcoding Kit (SQK-RBK004; Oxford Nanopore), sequenced on a R9.4 flow cell using a MinION (Oxford Nanopore) and basecalling was performed using Guppy within MinKnow. Long reads were demultiplexed using Porechop (v0.2.4) [40] and assembled with corresponding short reads generated from the Illumina MiSeq using Unicycler (v0.4.7) [41]. Mash dist (mash v2.2) [42] was performed on all *M. sciuri* genomes available in NCBI to determine genomic distance estimation to that of the cluster strain; the ten isolates with the greatest genomic similarity were incorporated into core SNP analysis as outlined previously. Metadata from NCBI was annotated within the phylogeny (Additional file 1: Table S5).

### Statistical analysis

Statistical associations between clinical data (onset; EOS v LOS and outcome of sepsis; reported deceased v non reported deceased), phenotypic (MRSA) and genomic traits (ARG/ST/VF) were explored for *S. aureus* using Chi-Square (χ^2^) tests, comparing means T test and the Log Rank test SPSS (version 25.0.0.1)*.* Kaplan–Meier survival curves were produced using the outcome follow up data (up to 60 days, or the last follow up record was used) in SPSS (version 25.0.0.1). The presence/absence of *S. aureus* VFs were analysed according to clinical onset and outcome of sepsis. Firstly, VFs associated with sepsis [43] were counted to provide a tailored VF score and a Kaplan–Meier survival curve was produced using the presence of Panton-Valentine Leukocidin (PVL), chosen as a frequently used clinical marker of *S. aureus* virulence. In addition, the frequency of all VFs detected > 98% identity and coverage from VFDB was counted against the clinical onset and outcome of sepsis to look for trends between EOS/LOS and alive/deceased. Statistical significance was taken at P value ≤ 0.05, and the false discovery rate was controlled using the Benjamini–Hochberg procedure [44]. A presence/absence matrix of all VFs detected per isolate (output from ABRicate vfdb) was generated using *splist2presabs* in the *fuzzysim* package in R 3.6.2.

## Results

### Characteristics of Gram-positive blood cultures reported during BARNARDS

In total, 1,266 GPB sepsis isolates were recorded from 1,239 neonates compared to 1,038 GNB sepsis isolates recorded from 1,007 neonates (Additional file 1: Table S6, Additional file 1: Fig. S2 [16]). From 296 GPB isolates available for identification, we identified 14 different species (Additional file 1: Fig. S3) and of these, 271 isolates were *Staphylococci* with 101 *Staphylococcus aureus* and 170 CoNS. Twenty-six isolates were identified as *Staphylococci sciuri,* but this species has since been reclassified to *Mammaliicoccus sciuri.* Overall, the onset of sepsis was 36% (n = 62) EOS, 54% (n = 71) LOS and for 10% (n = 17) cases the onset was undetermined (Additional file 1: Fig. S4). For *S. aureus,* the onset was 40% (n = 40) EOS, 47% (n = 47) LOS and undetermined for 13% (n = 14, Additional file 1: Fig. S4). Both *S. epidermidis* and *S. haemolyticus* was more common in LOS whereas *M. sciuri* was more common in EOS (Additional file 1: Fig. S4), however this data is indicative only.

This study specifically reports the antimicrobial susceptibility profiles of 144 isolates (n = 100 *S. aureus* and n = 44 CoNS) and WGS of 130 *Staphylococci/Mammaliicocci* sepsis isolates, (n = 100 *S. aureus* and n = 30 CoNS) which we were able to recover for microbiology characterisation after storage at − 80 °C.

### *Staphylococcus aureus* and CoNS phylogeny

**Fig. 1:**
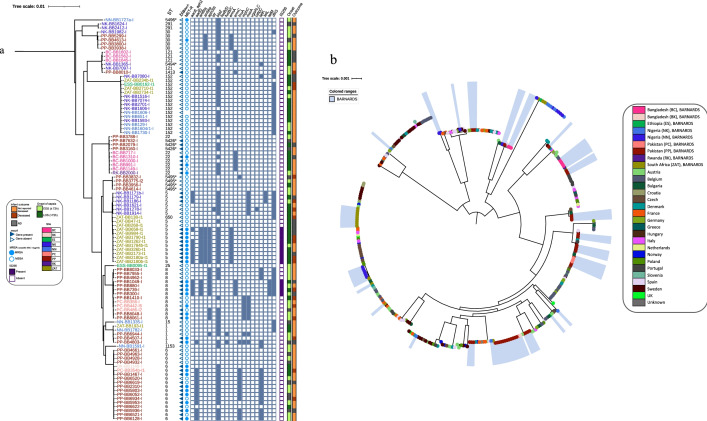
Core genome phylogenetic analysis of *Staphylococcus aureus including a global contextual analysis*. **a** Detailed core genome characterisation of 100 *Staphylococcus aureus* isolates (BARNARDS) using Roary (v3.12.0) and Fasttree (v2.1.11). Isolate labels are coloured according to clinical site of origin. The *in-silico* sequence type (ST) is shown outside of the isolate code (leaf). Presence of *mecA*, and whether the isolate was classified phenotypically as MRSA (as inferred from oxacillin MIC > 2 mg/l) is denoted by a filled triangle and/or circle respectively. Presence of IS*256* is denoted by a filled rectangle. **b** Core genome characterisation of 351 *Staphylococcus aureus* isolates, incorporating a European collection [29] using Roary (v3.12.0) and Fasttree (v2.1.11). Coloured ranges in blue represent a *S. aureus* from the BARNARDS collection. Branch labels are coloured according to country of origin. Symbol represents source of isolate

**Fig. 2:**
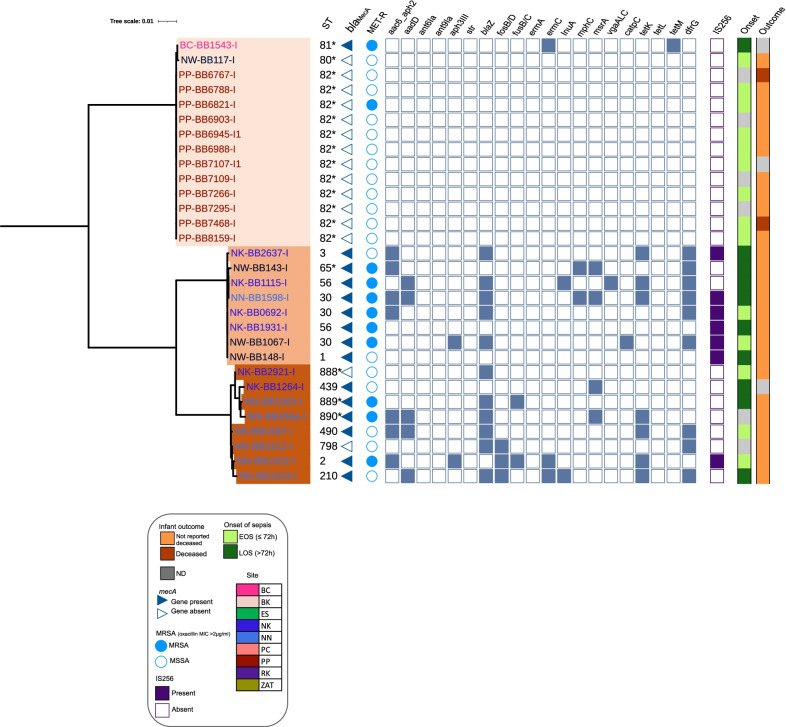
Core genome phylogenetic analysis of coagulase negative *Staphylococcus blood culture isolates*. Core genome phylogeny of CoNS isolates displaying key genomic traits for comparison using Roary (v3.12.0) and Fasttree (v2.1.11). Isolate labels are coloured according to clinical site of origin. Clades are highlighted according to species. The *in-silico* sequence type (ST) is shown outside of the isolate code (leaf). Presence of *mecA*, and whether the isolate was classified phenotypically as MRSA (as inferred from oxacillin MIC > 2 mg/l) is denoted by a filled triangle and/or circle respectively. Presence of IS*256* is denoted by a filled rectangle

**Fig. 3:**
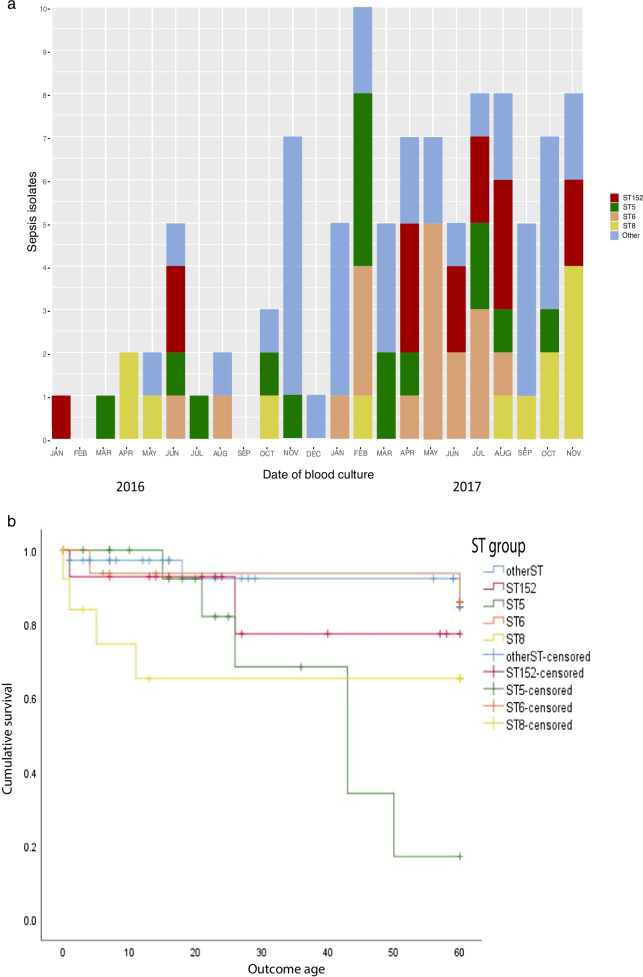
Temporal frequency and survival curve data for *Staphylococcus aureus* blood culture isolates. **a** Stacked bar graph to show the temporal frequency of *S. aureus* isolates recovered from blood cultures during the BARNARDS sampling, per month. The bar graph is coloured according to the dominant STs, with all other STs being grouped as ‘Other’. **b** A Kaplan Meier survival plot comparing the four dominant ST groups (with all other STs detected grouped together as a single group called ‘other’) against the age at outcome for the neonate and up to 60 days. Findings are suggestive as the data presented is from a sample size (ST152 n = 15, ST other n = 37, ST5 n = 17, ST6 n = 18 and ST8 n = 13; overall comparison P 0.041)

*S. aureus* for WGS characterisation included isolates predominantly from Pakistan (n = 42), Nigeria (n = 26), and South Africa (n = 16), along with nine from Bangladesh, two from Ethiopia and one from Rwanda, (n = 100 in total). In total, we detected 18 different sequence types (ST) (Fig. 1a) including the assignment of four novel STs; ST5426, ST5494, ST5945 and ST5496. We detected seven *S. aureus* clonal complex ST22 (CC22) from Bangladesh (n = 5), Pakistan (n = 1) and Rwanda (n = 1) and three ST5426 (within CC22) from Pakistan. Within BARNARDS, the commonest clonal complexes were CC5 (n = 36; 36%, consisting of ST6 [n = 18], ST5 [n = 17], ST650 [n = 1]) and CC8 (n = 13; 13%, all isolates belonged to ST8). Comparative genomic analyses including a previously known European collection, revealed ST5 isolates were distributed along 16 countries 45 (Fig. 1b). Interestingly, ST152 (n = 15) which is not assigned to a CC was the third most common ST identified. These isolates collectively represented 64% of the dataset and were isolated across the sampling period (Fig. 1a, Fig. 3a). All seventeen ST5 (Nigeria n = 6; South Africa, n = 11) were found carrying *mecA* (n = 12; 71%; Nigeria n = 3, South Africa n = 9; Fig. 2a) and the Nigerian and South African isolates grouped on different clades (Additional file 1: Fig. S5). Nine ST5 isolates collected from distinct South African neonates between July 2016 and July 2017 were within 10 pairwise SNPs (Supplementary Fig. 5), all were MRSA *spa* type t045, contained IS*256* and virulence factors *hlg, scn, spa* and *hly*. Mortality following biological sepsis (MfBS) was reported for four out of these nine neonates. Additionally, 12 ST152 were isolated from African sites (Ethiopia n = 1, Nigeria n = 10 and South Africa n = 1, Fig. 1a, Additional file 1: Fig. S6) and were MSSA.

ST6 (CC5) and ST8 (CC8) were found in South Asia (ST6 Pakistan n = 17/18, Bangladesh n = 1/18 and ST8 Pakistan n = 13, Fig. 1b, Supplementary Figs. 7–8). In the species-wide phylogeny, ST8 was found in 11 European countries, most notably Belgium and France (Fig. 1b). Within BARNARDS we saw three major ST8 clades, of which two were predominantly MRSA. (Fig. 1a, Supplementary Fig. 8). All ST8 *spa* type t064 genomes (n = 4/13) contained IS*256* and genes coding for VFs associated with leukocyte targeting (*Hlg*) and toxin production (*Hla*). All 18 ST6 isolates were *spa* type t304, regardless of the clinical site of origin or whether they were MRSA (Additional file 1: Fig. S8). Twelve of the 17 ST6 *S. aureus* from Pakistan collected between August 2016-July 2017 grouped within 10 SNPs. Overall, we found a significant difference between neonatal survival and ST type (χ^2^, P 0.016) and a Kaplan survival curve analysis displaying patient survival for the four most dominant STs (ST5, ST6, ST8, ST152) shows ST5 and ST8 *S. aureus* being more likely to result in a fatal outcome (Fig. 3b). Of note, two neonates with a ST5 *S. aureus* sepsis from South Africa and two neonates with a ST8 *S. aureus* sepsis from Pakistan were also culture positive for at least one GNB isolate from the same blood sample and were excluded from the survival curve analysis.

A total of 30 CoNS were characterised by WGS (Fig. 2), *S. epidermidis* (n = 8)*, M. sciuri* (n = 14) and *S. haemolyticus* (n = 8). The majority of *S. epidermidis* and *S. haemolyticus* were from Nigeria (NK n = 6, NN n = 7, NW n = 3), whereas n = 13/14 M*. sciuri* were from Asia, 12 being from one site in Pakistan (PP) and one from Bangladesh (BC). A single *M. sciuri* was recovered for WGS from Nigeria (NW). We found five STs from eight *S. haemolyticus* isolates including a novel assignment ST65 (Fig. 2). The most prominent ST (n = 3) found from all three sites in Nigeria was ST30, and all contained IS*256*. In n = 6/8 *S. haemolyticus* isolates IS*256* was detected (Fig. 2). When screening public databases, we detected five ST30 strains, of which one was a sepsis case from India (Additional file 1: Fig. S9). ST30 isolates within this study were at least 150 SNPs different, and further distinct to the Indian sepsis isolate deposited within NCBI. We detected one ST1 and one ST3 *S. haemolyticus.* There are currently 44 ST1 and 49 ST3 *S. haemolyticus* submitted to NCBI and associated metadata reveals that n = 20/44 ST1 and n = 21/49 ST3 caused sepsis, largely within Europe, however there were sporadic entries from Russia, Brazil, India, and USA (Additional file 1: Figs. S10 and S11). ST56 was only submitted once into NCBI (up to October 2020), and this originated from a non-clinical sample from Germany in 2016. Here we report two ST56 isolates from blood cultures collected in Nigeria, both were collected from the same clinical site (NK), both were resistant to methicillin (Fig. 2) and had 707 pairwise SNPs.

We found that all 8 *S. epidermidis* isolates belonged to different STs, including the assignment of three novel STs (ST888-890), of which two (ST889 and ST890) were methicillin resistant (Fig. 2). IS*256* was found in the ST2 *S. epidermidis* isolate from NN, Nigeria, however, was not found in any other isolates, although we present a limited dataset with available genomics data. ST490 was only detected once in the NCBI collection and was collected from a human sample. ST439 was detected twice, and an isolate from 2013 caused neonatal sepsis in the UK (ASM96671v1). Similarly, there were rare reports of ST210 within the NCBI collection (n = 5), and these were UK sepsis isolates from 2012 and 2017 (n = 3) and from human origin from the USA. ST2 was found in greater numbers in NCBI (n = 222), and the phylogeny separates into two main clades. The ST2 we report from Nigeria can be seen within the smaller clade alongside isolates from the environment, human commensal samples and those causing sepsis (Additional file 1: Fig. S12).

### Antimicrobial resistance

**Fig. 4:**
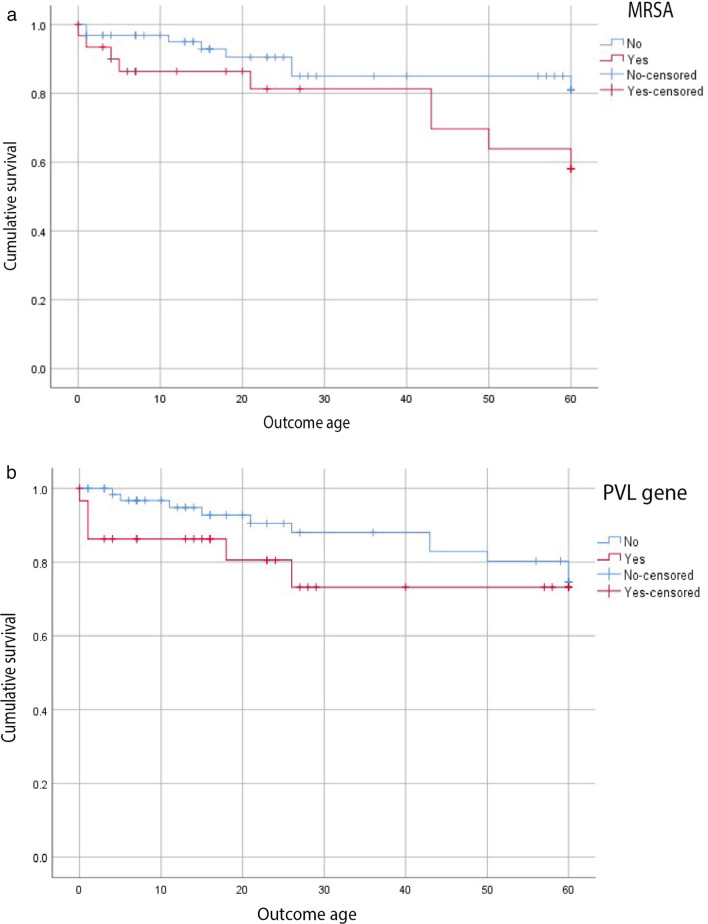
Neonatal survival curve data for *Staphylococcus aureus* and pathogenicity markers: MRSA and PVL. Kaplan Meier survival plot comparing **a** MRSA v MSSA and time to neonatal outcome, and **b** The presence of virulence factor PVL and time to neonatal outcome censored at the last available follow up appointment

In *S. aureus*, resistance rates were 53% (n = 49) to azithromycin, 32% (n = 32 to methicillin (oxacillin), 29% (n = 29) to tobramycin and 18% (n = 18) for gentamicin, (n = 100 isolates, except azithromycin n = 93; Table 1). Vancomycin and linezolid were the most active agents (0% resistance). The MIC_90_ of amikacin, tigecycline, minocycline, rifampicin, vancomycin and linezolid were all below the respective susceptible breakpoints (Table 1). Thirty-three were methicillin resistant *S. aureus* (MRSA), however the *mecA* gene was detected in 43% (n = 43). We also identified the reverse discrepancy, with three isolates negative for *mecA* (and *mecC*), but were classified MRSA by phenotypic methods. Typing of the staphylococcal cassette chromosome *mec* (SCC*mec*), revealed SCC*mec*_type_IVa(2B) was the most frequent (n = 30/43). All MRSA from Bangladesh carried SCC*mec*_type_IVa(2B) whereas all MRSA from South Africa carried SCC*mec*_type_IVa(1B). Although a 20% reduction in patient survival at 60 days was seen for neonates with MRSA compared to MSSA (Fig. 4a), this was not significant (Log Rank test, P 0.061), which may be due to the small sample size (n = 33). We found no significant difference between the onset of sepsis and MRSA (χ^2^, P 0.935 for phenotypic method and P 0.135 when *mecA* was used to denote MRSA).

**Table 1:** Antibiotic susceptibility testing for Gram-positive isolates recovered from blood cultures

		*S. aureus*					CoNS & *M. sciuri*		
		MIC(µg/mL)					MIC(µg/mL)		
Antibiotic class	Antimicrobial	Range	MIC50	MIC90	Resistance breakpoint	% Resistant	Range	Resistance breakpoint	% Resistant
Aminoglycosides	Amikacin	4- > 32	4	4	> 8	4.0	4– > 32	> 8	18.2
	Gentamicin	0.5– > 8	0.5	> 4	> 1	18.0	0.5– > 4	> 1	40.9
	Tobramycin	0.5– > 8	0.5	> 4	> 1	29.0	0.5– > 4	> 1	50.0
Fluoroquinolones	Ciprofloxacin	0.5– > 4	0.5	> 4	> 1	20.0	0.5– > 4	> 1	40.9
	Levofloxacin	0.5– > 4	0.5	> 4	> 1	15.0	0.5– > 4	> 1	40.9
Glycopeptides	Vancoymcin	1–2	1	1	> 2	0.0	1– > 8	> 4	2.3
MLS	Azithromycin	1– > 8	4	> 8	> 2	53.0	ND	ND	ND
Oxazolidinones	Linezolide	2–8	2	4	> 4	1.0	2– > 16	> 4	2.3
Ansamycins	Rifampicin	0.03	0.03	0.03	> 0.5	0.0	0.03– > 0.25	> 0.5	13.6
Penicillins	Ampicillin	2–128	NA	NA	NA	NA	2–64	NA	NA
	Flucloxacillin	0.5– > 8	NA	NA	NA	NA	1– > 8	NA	NA
	Oxacillin	1– > 8	1	> 8	> 2	33	1– > 8	0.25	100
Tetracyclines	Minocycline	0.25– > 2	0.25	0.5	> 0.5	7.0	0.25– > 2	> 0.5	34.1
	Tigecycline	0.25–0.5	0.5	0.5	> 0.5	0.0	0.25–4	> 0.5	20.5

Minimum inhibitory concentrations of antibiotics were determined by agar dilution. For *S. aureus* n = 100, except Azithromycin where n = 93. For CoNS and *M. sciuri* n = 44. Results interpreted according to EUCAST v11 guidelines and documents. The MIC_50_ and MIC_90_ were calculated for *S. aureus*

Although the MIC values indicate almost all *S. aureus* isolates were sensitive to both minocycline (Table 1; 93% [n = 93] sensitive) & tigecycline (100% [n = 100] sensitive), 25 isolates carried at least one tetracycline ARG (Fig. 2; n = 18 *tet*(K), n = 7 *tet*(M) and n = 3 *tet*(L). Additionally, aminoglycoside resistance genes were found in all *S. aureus*, and up to seven genes, including *aac*(3)*, aadD, sat4a* and *str,* were found in a cluster of isolates from South Africa (Fig. 1). The insertion element IS*256* is associated with the aminoglycoside resistance-mediating transposon Tn*4001*, and IS*256* was found in 13/100 isolates. Macrolide ARG (*ermA, ermC, InuA, mphC* and *msrA*) were predominantly found in South Asian isolates, including ST1, ST6, ST8, ST22 and ST121 *S. aureus.* We only detected macrolide ARG in ST5 isolates from Africa, where *msrA* genes were detected in isolates from Nigeria, and *ermA* genes were detected in ST5 from South Africa (Fig. 1a).

For CoNS, isolates’ resistance rates were 50% (n = 22) to tobramycin and 41% (n = 18) to gentamicin (Table 2). Fourteen isolates carried *bla*Z, 10 of which also carried at least one aminoglycoside ARG (*aadD* and *aph*(3) genes; Fig. 2). We also detected IS*256* in seven isolates (six *S. haemolyticus* and one *S. epidermidis*)*.* Resistance to methicillin in CoNS (MR-CoNS) was seen in n = 11/18 isolates of *S. epidermidis*, n = 9/11 of *S. haemolyticus* and n = 2/13 of *M. sciuri* (Table 1, Fig. 2). *mecA* was detected in an additional four isolates, however all were sensitive to oxacillin. All *M. sciuri* isolates had the *mecA1* gene, however this did not confer resistance to ß-lactam antibiotics, as reported previously 46. We detected two copies of *mecA* (*mecA1_2* and *mecA_9*) in one isolate from Bangladesh, ultimately rendering this isolate resistant to oxacillin with an MIC > 8 mg/l (Fig. 2, Table 1). Furthermore, a single *M. sciuri* isolate from Pakistan was resistant to methicillin, tigecycline and vancomycin. Aside from these two isolates, the remaining 13 M*. sciuri* tested were sensitive to all other antibiotics. We also found a single *vgaALC* and *catPC* gene in two *S. haemolyticus* isolates, both of which also carried *mecA*, and aminoglycoside resistance genes (*aadD* and *aph*(3)*-III)*.

**Table 2:** In silico* Staphylococcus aureus* virulence factors analysed for both onset and outcome of sepsis

		Onset				Outcome			
Function	VF	EOS	LOS	ND	Total	Alive	Deceased	ND	Total
Targeting leukocytes	*PVL*	10	19	1	30	17	6	7	30
	*lukD*	25	29	12	66	36	14	16	66
	*hlgABC*	40	46	14	100	62	18	20	100
Inhibiting host complement systems	*chp*	16	15	5	36	19	8	9	36
	*scn*	39	39	13	91	60	14	17	91
Death of B cells	*spa*	18	22	8	48	27	10	11	48
Formation of aggregates in blood	*coa*	3	3	2	8	8	0	0	8
	*clfB*	1	2	1	4	4	0	0	4
	*clfA*	10	5	1	16	12	3	1	16
	*vWbp*	10	5	2	17	12	3	2	17
*S. aureus* tether to endothelial cells	*fnbB*	3	3	1	7	6	0	1	7
	*fnbA*	5	8	0	13	12	0	1	13
Toxin expression causing injury to endothelium disrupting barrier	*hly/hla*	30	36	13	79	47	14	18	79

Virulence factors (VF) grouped into reported biological functions [43] and further delineated to show the frequency of *S. aureus* isolates containing VFs for both onset of sepsis (early onset [EOS] and late onset sepsis [LOS]) and neonatal outcome (not reported deceased from the latest available follow up date, and deceased). ND indicates an unknown onset and/or outcome of sepsis

### Virulence factors

From 100 *S. aureus* isolates, 5494 genes putatively related to virulence (> 98% coverage and identity) were detected in total. All VF hits were summarised according to the gene presence/absence against sepsis onset (EOS v LOS) and outcome (alive v deceased) extending on analysis reported in [46]. For example, for the gene *geh,* we saw 32/40 (80%) presence in EOS as opposed to 24/46 (52.2%) in LOS. For the six genes that were significant before FDR correction (*geh, cap8H, sak, cap8I, cap8K and scn*), all had 13% or greater presence for EOS than for LOS (Additional file 1: Table S7). Similarly, *lukD* was present in 16/18 (88.9%) of *S. aureus* isolates recovered from deceased neonates, but only in 50/82 (61%) of neonates recorded as alive at the latest follow up appointment/60 days. For the eight genes that were significant before FDR correction, (*lukD, sbi, aur, cap8B, scn, esaE, essC, hlgB),* all bar one had at least 19% greater gene presence in the deceased than the survivors (Additional file 1: Table S7). The exception being *scn* where we saw 14/18 (77.8%) in the deceased with 77/82 (93.9%) in the survivors. This analysis was performed on the presence/absence of single genes, and further work is needed to investigate the role of multiple VF in onset/outcome of sepsis.

We also grouped VF related to bloodstream invasion/infection (n = 656) according to their biological functions as described by Powers et al*.,* 2014 43 (Table 2, Additional file 1: Fig. S13). *hlgA/B/C* and *lukD* genes were found in 100% (n = 100) and 66% (n = 66) of *S. aureus* respectively, and PVL was found in 30% (n = 30). In neonates reported as deceased (n = 17), we found that the presence of PVL was found to significantly reduce the time to death (comparing means test, P 0.028), with the mean time to mortality at eight days compared to 31 days for PVL negative *S. aureus.* Similarly, the Kaplan Meier survival curve suggests the presence of PVL may result in a rapid MfBS, and this was more pronounced in the first 30 days of life, however, was not statistically significant (Log Rank test, P 0.251, Fig. 4b). In addition to virulence associated genes targeting leukocytes, we detected genes that contribute to inhibiting host complement systems (*chp,* and *scn*) and genes that contribute to *S. aureus* aggregation (Table 2, *coa* n = 8 isolates, *cflB* n = 4*, clfA* n = 16 and *vWbp* n = 17).

We found eight virulence associated genes from seven CoNS isolates. Two hits of the antiphagocytosis genes *cap8G* and *cap8E* within the same contig were detected in a *S. haemolyticus* isolate, whereas the remaining genes were detected within *S. epidermidis*. VF from the biofilm related *ica* operon (*icaA)* were detected in three *S. epidermidis* isolates, of which one contained IS*256* and was MRSA (from Nigeria National Hospital, Abuja (NN)), whereas *esxA,* a gene associated with bacterial survival (type VII secretion system), were found in three *S. epidermidis* isolates (NK n = 2, NN n = 1). VFs present in VFDB were not detected within *M. sciuri* isolates.

### *M. sciuri* cluster

**Fig. 5:**
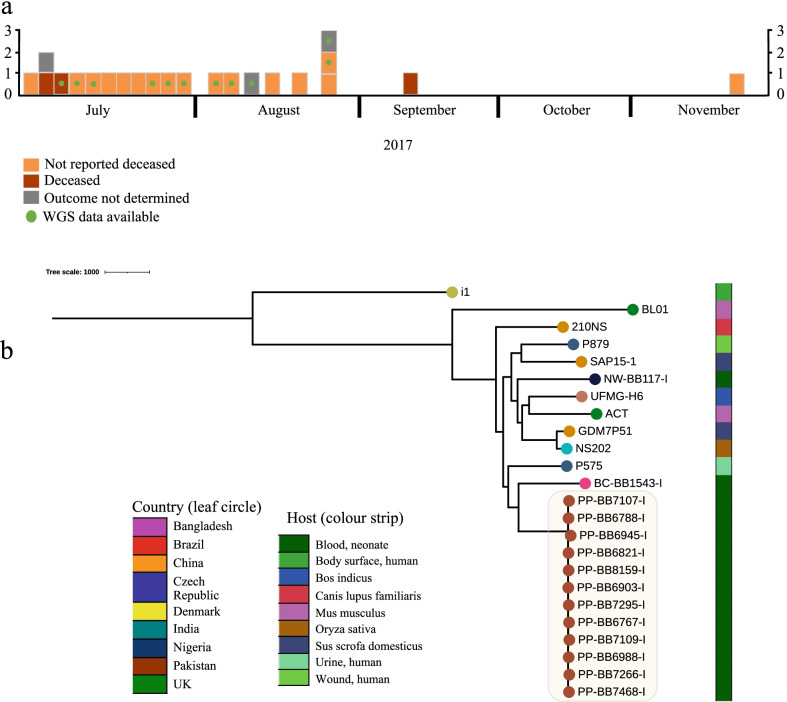
**a** Analysis of *Mammaliicoccus sciuri* recovered from blood cultures and a WGS contextual analysis. A timeline of *M. sciuri* neonatal sepsis in Pakistan, indicating which were available for whole genome characterisation. The blocks represent an individual case, and are colour coded according to the clinical outcome of sepsis. **b** A core genome SNP tree for all PP-BB isolates with WGS data available, performed using snippy-gubbins and Raxml (please refer to the text for details on methods) **c** Comparative phylogenetic tree of all *S. sciuri* with WGS data available in BARNARDS including the 10 genetically closest strains of *M. sciuri* from NCBI

Twenty-two *M. sciuri* were collected from Islamabad, Pakistan, between July-November 2017 (Fig. 5a). The blood culture date was not available for eight and we inferred approximate dates based on the consecutive enrolment numbers for the study (Fig. 5a). Where the onset of sepsis was known (n = 13), all were EOS suggesting the same strain may have spread within the hospital environment, perhaps related to the delivery area/delivery practices as, commonly reported, hospital contamination is more often linked to LOS [5, 47]. WGS data was available for 11 isolates which were all within four pairwise SNPs and were assigned to ST82, suggesting these represented the same/phylogenetically very close strains. The hybrid assembly of short and long read sequences produced a single contig for the bacterial chromosome at 2,932,999 bp (Additional file 1: Fig. S14). From genome estimation screening using mash (v2.2), the 10 genetically closest genomes to the Pakistani *M. sciuri* were > 1,400 SNPs away. The isolate that shares the same branch point in the collective phylogeny (Fig. 5b), was an *M. sciuri* blood culture isolate collected from Bangladesh during this study and was assigned to ST81. Similarly, the single *M. sciuri* isolate available for WGS characterisation from Nigeria was assigned to ST80. The MLST scheme for *M. sciuri* was first made available within pubmlst.org on the 1^st^ of February 2021. Of the *M. sciuri* genomes that had the greatest Jaccard similarity index and were included into the phylogenetic analysis, three were clinical samples from Europe (Denmark and Czech Republic) and these were distinct throughout the tree suggesting different lineages causing human infection.

## Discussion

When a neonate is presenting with clinical signs of sepsis, empirical antibiotic treatment will be prescribed. Often, in LMICs hospitals there are not sufficient resources to perform necessary laboratory diagnostics, or a confirmatory blood culture. There is currently little to no data extending beyond an initial species identification (often via Gram staining and biochemical tests) and an antibiogram when Staphylococci/GPB are reported from neonatal blood cultures. We performed this retrospective study to gain an insight into the diversity of GPB isolated from blood cultures, and we highlight the need for future genomic and epidemiological studies to uncover the true burden of GPB neonatal sepsis in LMICs. A recent review by Garcia et al., 2022 describe a “Paucity of [care] bundles and elements aimed at HCAI detection (4% of 295 elements)” and “barriers to strengthening laboratory capacity”[48] when addressing matters of infection control in neonatal care in LMICs, emphasising the need for surveillance studies also mirrored elsewhere [49].

*S. aureus* is often the most frequently identified Gram-positive cause of neonatal sepsis in LMICs [2, 7, 50]. One of the most common STs found in this study was ST152, and although ST152 is not associated to a particular CC, scanning the list on the PubMLST database isolate collection [24], we noticed that of the 40 isolates with this ST, 12 were collected from blood cultures and all were from Africa (n = 9 The Gambia and n = 3 Mozambique). Herein, we found ST152 *S. aureus* neonatal sepsis in Ethiopia, Nigeria, and South Africa. Additionally, we detected a cluster of ST5 (CC5) in South Africa and previous published work from the same hospital suggests that ST5 is a successful and problematic lineage [51]. Many countries have reported CC5 MRSA from routine hospital molecular surveillance causing nosocomial infections further suggesting its global importance [51–56]. Frequently, however, nosocomial epidemiological clusters or outbreaks go undetected perhaps due to limited resources and/or protocols in place to detect outbreaks, outlining the importance of genomic surveillance studies to tackle nosocomial infection and provide insight into transmission dynamics of bacteria within neonatal units. Similar to all ST152 *S. aureus* being found only in Africa, during this study, all ST8 isolates were from neonates in South Asia. The numerous clades within the *S. aureus* ST8 group (as inferred from SNP analysis of the ST8 reads) suggest multiple introductions of ST8 causing neonatal sepsis in Pakistan. CC8 is well established and has commonly been reported from clinical infections worldwide, including South Asia [57, 58]. Importantly, we found that infection with ST8 and ST5 *S. aureus* had lower survival in the Kaplan Meir curve compared to other ST groups suggesting that certain lineages of *S. aureus* may be more pathogenic and problematic in neonatal sepsis in LMICs. Previous work on *S. aureus* bacteraemia also suggests that clonal differences may be a contributing indicator of infection outcome [59], and through preliminary data analysis we indicate that certain STs, ARGs, MGEs and VFs may be used as indicators of neonatal outcome.

Although *S. aureus* neonatal sepsis isolates in this study remained susceptible to vancomycin, similar to many previous reports [8, 60], we report an MRSA rate of 33% slightly higher than reported in a single site study in Uganda [61], and similar to a study in Nigeria [62], emphasising MRSA as an important source of infection and further highlighting the burden of AMR in neonatal sepsis in LMICs. Importantly, CoNS isolates often carry methicillin resistant genes [9, 13, 15], and through WGS and MIC determination, we identified methicillin resistance in one-third of isolates analysed, highest in *S. epidermidis* (n = 6/8) and *S. haemolyticus* (n = 7/8). Meric et al*.* [10] showed the importance of horizontal gene transfer events to increase the likelihood of divergent lineages causing infection due to the acquisition of genetic traits (virulence genes and ARG, e.g. SCC*mecA*) potentially conferring niche adaptations associated with pathogenicity. Kozitskaya et al.[63] also reports a significant association between the presence of IS*256* in clinical *S. epidermidis*, perhaps suggesting that isolates with increased antimicrobial resistance are successfully colonising hospital environments. Although our WGS dataset for CoNS is too small for such comparisons, and found relatively few IS*256* in *S. epidermidis*, we have shown concordance with previous studies and published genomics data, indicating that further surveillance studies are needed to characterise GPB isolates recovered from neonatal blood cultures.

The biggest question raised upon the isolation of CoNS from blood cultures is whether this confirms biological sepsis or represents contamination from transient colonisation of the skin. The ANISA study classified all CoNS as “definite contaminants”[64] and CoNS were likewise excluded from Okomo et al.*’s*.,[2] meta-analysis, many neonatal studies report CoNS as a leading cause of neonatal sepsis [65–67]. Our results must be interpreted with this in mind. A second blood culture is common practice following the identification of CoNS, however this remains challenging in neonates, as often a small volume of blood was collected (volume not recorded), and it was not possible for sites to consistently perform duplicate blood cultures following the local species identification of a CoNS. Speciation of CoNS can be difficult in laboratories where biochemical and conventional microbiology techniques are routinely employed [9], and long-term improvements in access to microbiology diagnostics in LMICs will be vital to understand the true burden of Staphylococci neonatal sepsis in these sites. Without this capacity, the cluster of *M. sciuri* described here could not be recognised and managed, as one clear example of the value of diagnostic capacity. *M. sciuri* have a broad range of habitats including animals, humans and the environment [45]. During a recent phylogenomic comparison of *Staphylococci,* several CoNS species were reclassified including *Staphylococcus sciuri* [68]*.* Although infection caused by *M. sciuri* has largely been reported in animals, there are case reports of *M. sciuri* causing peritonitis [69] and two distinct cases of catheter associated sepsis in Turkey [70] and Japan [71]. The clinical and microbiology team in Pakistan were routinely reporting and storing all GPB isolates from blood cultures, in contrast to other sites in South Asia which is likely to account for the steep difference in GPB neonatal sepsis, and the majority (n = 21, 81%) of *M. sciuri* isolates from Pakistan (Fig. 5). We did however identify five *M. sciuri* isolates from other clinical sites in Bangladesh, Ethiopia, and Nigeria. From these, two isolates (from Bangladesh and Nigeria) were shown to be sitting on separate phylogenetic branches through genomics analysis, (Fig. 5b). Furthermore, the identification of *M. sciuri* from 5/12 clinical sites highlights the potential for uncommon pathogens to be present in clusters or cause outbreaks.

There are limitations to this study. BARNARDS was established to determine the aetiology of GNB sepsis, therefore there was heterogeneity in the reporting and storage of GPB isolates among the clinical sites. Although many clinical sites that participated in BARNARDS identified GPB species from blood cultures as part of their routine standard operating procedures and guided by local practices, not all sites had the capacity and resources to identify bacteria to the species level. The retrospective recovery of Gram-positive isolates at local sites was greatest in Nigeria and Pakistan, therefore caution must be taken in any interpretation of results. There was discrepancy between the degree of reporting GPB neonatal sepsis within the clinical sites therefore we make no reference to prevalence or specific country analyses within this dataset. All statistical analyses between clinical data and microbiology findings are exploratory and hypothesis forming. Group B *Streptococci* (GBS), considered the dominant cause of sepsis in high-income countries [47, 72], was not found/reported, and this has also been observed in other studies [1, 5, 6, 8, 60, 73]. With limited microbiology resources in many LMICs however (GBS require additional media and growth requirements), septic neonates may be deceased prior to a blood culture and Vera et al*.,* suggest GBS are perhaps underreported due to these infections presenting very early into life [47]. Additionally, due to the nature of our retrospective recovery, the loss of fastidious organisms at the local sites and/or transportation to the UK might have hindered the recovery of GBS and further studies are needed to determine the incidence of *Staphylococci* and GBS neonatal sepsis.

## Conclusion

*S. aureus* is a leading cause of neonatal sepsis in LMICs, and we found one third of *S. aureus* to be MRSA. Future studies should assess the use of genomic characterisation to predict whether certain lineages of Staphylococci, and GPB species have greater pathogenic potential in neonatal sepsis. Unveiling a cluster of *M. sciuri* in Pakistan further demonstrates the importance of GPB in neonatal sepsis. There is an urgent need for microbiological and genomic surveillance studies with strict infection control practices to categorically evaluate the true burden of GPB in neonatal sepsis in LMICs, laying emphasis on whether associations exist between certain species, lineages and virulence factors and the clinical onset or outcome of sepsis.

## Supplementary Information

**Additional file 1. **Additional tables and figures.

## Data Availability

Sequences have been deposited in ENA with the project number: PRJEB40908, with individual accessions reported in the supplementary dataset (Additional file 1: Table S3). The *M. sciuri* genome used as a reference for SNP calling has been deposited in Genbank with the accession number CP071138.

## Abbreviations

AMR: Antimicrobial resistance
ARG: Antibiotic resistance genes
ATCC: American Type Culture Collection
BARNARDS: Burden of Antibiotic Resistance in Neonates from Developing Societies
CC: Clonal complex
CoNS: Coagulase negative Staphylococci
EOS: Early onset sepsis
EUCAST: European Committee on Antimicrobial Susceptibility Testing
GNB: Gram negative bacteria
GPB: Gram positive bacteria
IS: Insertion sequence
LMIC: Low- and- middle income country
LOS: Late onset sepsis
MfBs: Mortality following biological sepsis
MGE: Mobile genetic element
MIC: Minimum inhibitory concentration
MLST: Multi locus sequence typing
MRSA: Methicillin resistant *Staphylococcus aureus*
PVL: Panton-Valentine Leukocidin
SNP: Single nucleotide polymorphism
ST: Sequence type
VF: Virulence factors
VFDB: Virulence factor database
VLBW: Very low birth weight
WGS: Whole genome sequencing
