# Supplementary material for: Characterisation of Staphylococci species from neonatal blood cultures in low- and middle-income countries
Source: BMC Infect Dis. 2022 Jul 1;22:593. doi: 10.1186/s12879-022-07541-w (PMC9254428; doi:10.1186/s12879-022-07541-w)
Supplement: Supplementary file 1 — Additional file 1. Additional tables and figures. [file 12879_2022_7541_MOESM1_ESM.docx]

Additional file 1: Table S1 – Ethical approval for each clinical site

| **Clinical site** | **Committees** | **Principal Investigator** | **Reference(s)** | **Approval date(s)** |
| --- | --- | --- | --- | --- |
| **BC** | Ethical Review Committee, Bangladesh Institute of Child Health | Samir Kumar Saha | BICH-ERC-4/3/2015 | 15/09/2015 |
| **BK** | Ethical Review Committee, Bangladesh Institute of Child Health | Samir Kumar Saha | BICH-ERC-4/3/2015 | 15/09/2015 |
| **ES** | Boston Children's Hospital | Grace Chan | IRB-P00023058 | 11/08/2016 |
| **IN** | Institutional Ethics Committee, National Institute of Cholera and Enteric Diseases and Institue of Post Graduate Medical Education and Research, IPGME&R Research Oversight Committee | Sulagna Basu | A-I/2016-IEC and Inst/IEC/2016/508 | 17/11/2016 and 04/11/2016 |
| **NK** | Kano State Hospitals Management Board | Kenneth Iregbu | 8/10/1437AH | 13/07/2016 |
| **NN** | Health Research Ethics Committee (HREC), National Hospital, Abuja | Kenneth Iregbu | NHA/EC/017/2015 | 27/04/2015 |
| **NW** | Health Research Ethics Committee (HREC), National Hospital, Abuja | Kenneth Iregbu | NHA/EC/017/2015 | 27/04/2015 |
| **PC** | Shaheed Zulfiqar Ali Bhutto Medical University, Pakistan Institute of Medical Sciences (PIMS) Islamabad | Rabaab Zahra | NA, signed letter from Prof. Tabish Hazir | 27/05/2015 |
| **PP** | Shaheed Zulfiqar Ali Bhutto Medical University, Pakistan Institute of Medical Sciences (PIMS) Islamabad | Rabaab Zahra | NA, signed letter from Prof. Tabish Hazir | 27/05/2015 |
| **RK** | Republic of Rwanda, National Ethics Committee | Jean-Baptiste Mazarati | No342/RNEC/2015 | 10/11/2015 |
| **RU** | Republic of Rwanda, National Ethics Committee | Jean-Baptiste Mazarati | No342/RNEC/2015 | 10/11/2015 |
| **ZAT** | Stellenbosch University and Tygerberg Hospital, Research projects, Western Cape Government | Shaheen Mehtar | N15/07/063 | 04/12/2015 and 02/02/2016 |

Additional file 1: Table S2 – The antibiotics and concentrations used for the antimicrobial susceptibility testing.

Additional file 1: Table S3 – List of all accessions for isolates in this study.

| BBisolate | ENA experiment | ENA run | ENA accession |
| --- | --- | --- | --- |
| BC-BB1000-I | ERX4858602 | ERR5052487 | ERS5229019 (SAMEA7471323) |
| BC-BB1149-I | ERX4858603 | ERR5052488 | ERS5229020 (SAMEA7471324) |
| BC-BB1310-I | ERX4858604 | ERR5052489 | ERS5229021 (SAMEA7471325) |
| BC-BB1562-I | ERX4858605 | ERR5052490 | ERS5229022 (SAMEA7471326) |
| BC-BB1602-I | ERX4858606 | ERR5052491 | ERS5229023 (SAMEA7471327) |
| BC-BB1645-I | ERX4858607 | ERR5052492 | ERS5229024 (SAMEA7471328) |
| BC-BB717-I | ERX4858608 | ERR5052493 | ERS5229025 (SAMEA7471329) |
| BC-BB991-I | ERX4858609 | ERR5052494 | ERS5229026 (SAMEA7471330) |
| BK-BB1140-I | ERX4858610 | ERR5052495 | ERS5229027 (SAMEA7471331) |
| ESS-BB0095-I1 | ERX4858611 | ERR5052496 | ERS5229028 (SAMEA7471332) |
| ESS-BB0162-I1 | ERX4858612 | ERR5052497 | ERS5229029 (SAMEA7471333) |
| NK-BB1171b-I | ERX4858613 | ERR5052498 | ERS5229030 (SAMEA7471334) |
| NK-BB1179-I | ERX4858614 | ERR5052499 | ERS5229031 (SAMEA7471335) |
| NK-BB1186-I | ERX4858615 | ERR5052500 | ERS5229032 (SAMEA7471336) |
| NK-BB1278-I | ERX4858616 | ERR5052501 | ERS5229033 (SAMEA7471337) |
| NK-BB1365-I | ERX4858617 | ERR5052502 | ERS5229034 (SAMEA7471338) |
| NK-BB1516-I | ERX4858618 | ERR5052503 | ERS5229035 (SAMEA7471339) |
| NK-BB1593-I | ERX4858619 | ERR5052504 | ERS5229036 (SAMEA7471340) |
| NK-BB1606-I | ERX4858620 | ERR5052505 | ERS5229037 (SAMEA7471341) |
| NK-BB1621-I | ERX4858621 | ERR5052506 | ERS5229038 (SAMEA7471342) |
| NK-BB1624-I | ERX4858622 | ERR5052507 | ERS5229039 (SAMEA7471343) |
| NK-BB1914-I | ERX4858623 | ERR5052508 | ERS5229040 (SAMEA7471344) |
| NK-BB1982-I | ERX4858624 | ERR5052509 | ERS5229041 (SAMEA7471345) |
| NK-BB2412-I | ERX4858625 | ERR5052510 | ERS5229042 (SAMEA7471346) |
| NK-BB2701-I | ERX4858626 | ERR5052511 | ERS5229043 (SAMEA7471347) |
| NK-BB7074-I | ERX4858627 | ERR5052512 | ERS5229044 (SAMEA7471348) |
| NK-BB7080-I | ERX4858628 | ERR5052513 | ERS5229045 (SAMEA7471349) |
| NK-BB7097-I | ERX4858629 | ERR5052514 | ERS5229046 (SAMEA7471350) |
| NN-BB129-I | ERX4858630 | ERR5052515 | ERS5229047 (SAMEA7471351) |
| NN-BB1335-I | ERX4858631 | ERR5052516 | ERS5229048 (SAMEA7471352) |
| NN-BB1591-I | ERX4858632 | ERR5052517 | ERS5229049 (SAMEA7471353) |
| NN-BB1604r1-I | ERX4858633 | ERR5052518 | ERS5229050 (SAMEA7471354) |
| NN-BB1606-I | ERX4858634 | ERR5052519 | ERS5229051 (SAMEA7471355) |
| NN-BB1727a-I | ERX4858635 | ERR5052520 | ERS5229052 (SAMEA7471356) |
| NN-BB1730-I | ERX4858636 | ERR5052521 | ERS5229053 (SAMEA7471357) |
| NN-BB1782-I | ERX4858637 | ERR5052522 | ERS5229054 (SAMEA7471358) |
| NN-BB651-I | ERX4858638 | ERR5052523 | ERS5229055 (SAMEA7471359) |
| PC-BB354b-I1 | ERX4858639 | ERR5052524 | ERS5229056 (SAMEA7471360) |
| PC-BB356-I | ERX4858640 | ERR5052525 | ERS5229057 (SAMEA7471361) |
| PC-BB442-I5 | ERX4858641 | ERR5052526 | ERS5229058 (SAMEA7471362) |
| PC-BB486-I2 | ERX4858642 | ERR5052527 | ERS5229059 (SAMEA7471363) |
| PP-BB1049-I | ERX4858643 | ERR5052528 | ERS5229060 (SAMEA7471364) |
| PP-BB1410-I | ERX4858644 | ERR5052529 | ERS5229061 (SAMEA7471365) |
| PP-BB1467-I | ERX4858645 | ERR5052530 | ERS5229062 (SAMEA7471366) |
| PP-BB2079-I | ERX4858646 | ERR5052531 | ERS5229063 (SAMEA7471367) |
| PP-BB2310-I | ERX4858647 | ERR5052532 | ERS5229064 (SAMEA7471368) |
| PP-BB300-I | ERX4858648 | ERR5052533 | ERS5229065 (SAMEA7471369) |
| PP-BB3160-I | ERX4858649 | ERR5052534 | ERS5229066 (SAMEA7471370) |
| PP-BB3775-I2 | ERX4858650 | ERR5052535 | ERS5229067 (SAMEA7471371) |
| PP-BB3788-I | ERX4858651 | ERR5052536 | ERS5229068 (SAMEA7471372) |
| PP-BB3800-I | ERX4858652 | ERR5052537 | ERS5229069 (SAMEA7471373) |
| PP-BB3832-I | ERX4858653 | ERR5052538 | ERS5229070 (SAMEA7471374) |
| PP-BB3938-I | ERX4858654 | ERR5052539 | ERS5229071 (SAMEA7471375) |
| PP-BB3956-I | ERX4858655 | ERR5052540 | ERS5229072 (SAMEA7471376) |
| PP-BB4507-I | ERX4858656 | ERR5052541 | ERS5229073 (SAMEA7471377) |
| PP-BB4613-I | ERX4858657 | ERR5052542 | ERS5229074 (SAMEA7471378) |
| PP-BB4614-I | ERX4858658 | ERR5052543 | ERS5229075 (SAMEA7471379) |
| PP-BB4661-I | ERX4858659 | ERR5052544 | ERS5229076 (SAMEA7471380) |
| PP-BB4803-I | ERX4858660 | ERR5052545 | ERS5229077 (SAMEA7471381) |
| PP-BB4928-I | ERX4858661 | ERR5052546 | ERS5229078 (SAMEA7471382) |
| PP-BB4932-I | ERX4858662 | ERR5052547 | ERS5229079 (SAMEA7471383) |
| PP-BB4962-I | ERX4858663 | ERR5052548 | ERS5229080 (SAMEA7471384) |
| PP-BB4963-I | ERX4858664 | ERR5052549 | ERS5229081 (SAMEA7471385) |
| PP-BB5299-I | ERX4858665 | ERR5052550 | ERS5229082 (SAMEA7471386) |
| PP-BB5803-I | ERX4858666 | ERR5052551 | ERS5229083 (SAMEA7471387) |
| PP-BB5936-I | ERX4858667 | ERR5052552 | ERS5229084 (SAMEA7471388) |
| PP-BB5953-I | ERX4858668 | ERR5052553 | ERS5229085 (SAMEA7471389) |
| PP-BB6052-I | ERX4858669 | ERR5052554 | ERS5229086 (SAMEA7471390) |
| PP-BB6128-I | ERX4858670 | ERR5052555 | ERS5229087 (SAMEA7471391) |
| PP-BB6520-I | ERX4858671 | ERR5052556 | ERS5229088 (SAMEA7471392) |
| PP-BB6521-I | ERX4858672 | ERR5052557 | ERS5229089 (SAMEA7471393) |
| PP-BB6619-I | ERX4858673 | ERR5052558 | ERS5229090 (SAMEA7471394) |
| PP-BB6622-I | ERX4858674 | ERR5052559 | ERS5229091 (SAMEA7471395) |
| PP-BB6934-I | ERX4858675 | ERR5052560 | ERS5229092 (SAMEA7471396) |
| PP-BB6944-I | ERX4858676 | ERR5052561 | ERS5229093 (SAMEA7471397) |
| PP-BB739-I | ERX4858677 | ERR5052562 | ERS5229094 (SAMEA7471398) |
| PP-BB7632-I | ERX4858678 | ERR5052563 | ERS5229095 (SAMEA7471399) |
| PP-BB7955-I | ERX4858679 | ERR5052564 | ERS5229096 (SAMEA7471400) |
| PP-BB8010-I | ERX4858680 | ERR5052565 | ERS5229097 (SAMEA7471401) |
| PP-BB8033-I | ERX4858681 | ERR5052566 | ERS5229098 (SAMEA7471402) |
| PP-BB8048-I | ERX4858682 | ERR5052567 | ERS5229099 (SAMEA7471403) |
| PP-BB8061-I | ERX4858683 | ERR5052568 | ERS5229100 (SAMEA7471404) |
| PP-BB880-I | ERX4858684 | ERR5052569 | ERS5229101 (SAMEA7471405) |
| RK-BB2000-I | ERX4858685 | ERR5052570 | ERS5229102 (SAMEA7471406) |
| ZAT-BB1262-I1 | ERX4858686 | ERR5052571 | ERS5229103 (SAMEA7471407) |
| ZAT-BB138-I1 | ERX4858687 | ERR5052572 | ERS5229104 (SAMEA7471408) |
| ZAT-BB1784b-I1 | ERX4858688 | ERR5052573 | ERS5229105 (SAMEA7471409) |
| ZAT-BB1790-I1 | ERX4858689 | ERR5052574 | ERS5229106 (SAMEA7471410) |
| ZAT-BB193-I1 | ERX4858690 | ERR5052575 | ERS5229107 (SAMEA7471411) |
| ZAT-BB2173-I1 | ERX4858691 | ERR5052576 | ERS5229108 (SAMEA7471412) |
| ZAT-BB2180a-I1 | ERX4858692 | ERR5052577 | ERS5229109 (SAMEA7471413) |
| ZAT-BB2180b-I1 | ERX4858693 | ERR5052578 | ERS5229110 (SAMEA7471414) |
| ZAT-BB234b-I1 | ERX4858694 | ERR5052579 | ERS5229111 (SAMEA7471415) |
| ZAT-BB268-I1 | ERX4858695 | ERR5052580 | ERS5229112 (SAMEA7471416) |
| ZAT-BB2710-I1 | ERX4858696 | ERR5052581 | ERS5229113 (SAMEA7471417) |
| ZAT-BB2734-I1 | ERX4858697 | ERR5052582 | ERS5229114 (SAMEA7471418) |
| ZAT-BB326b-I1 | ERX4858698 | ERR5052583 | ERS5229115 (SAMEA7471419) |
| ZAT-BB47-I1 | ERX4858699 | ERR5052584 | ERS5229116 (SAMEA7471420) |
| ZAT-BB658-I1 | ERX4858700 | ERR5052585 | ERS5229117 (SAMEA7471421) |
| ZAT-BB984-I1 | ERX4858701 | ERR5052586 | ERS5229118 (SAMEA7471422) |
| BC-BB1543-I | ERX4858702 | ERR5052587 | ERS5229119 (SAMEA7471423) |
| NK-BB0692-I | ERX4858703 | ERR5052588 | ERS5229134 (SAMEA7471438) |
| NK-BB1115-I | ERX4858704 | ERR5052589 | ERS5229135 (SAMEA7471439) |
| NK-BB1264-I | ERX4858705 | ERR5052590 | ERS5229141 (SAMEA7471445) |
| NK-BB1931-I | ERX4858706 | ERR5052591 | ERS5229136 (SAMEA7471440) |
| NK-BB2637-I | ERX4858707 | ERR5052592 | ERS5229133 (SAMEA7471437) |
| NK-BB2921-I | ERX4858708 | ERR5052593 | ERS5229142 (SAMEA7471446) |
| NN-BB1022-I | ERX4858709 | ERR5052594 | ERS5229143 (SAMEA7471447) |
| NN-BB1503-I | ERX4858710 | ERR5052595 | ERS5229147 (SAMEA7471451) |
| NN-BB1522-I | ERX4858711 | ERR5052596 | ERS5229144 (SAMEA7471448) |
| NN-BB1587-I | ERX4858712 | ERR5052597 | ERS5229145 (SAMEA7471449) |
| NN-BB1598-I | ERX4858713 | ERR5052598 | ERS5229137 (SAMEA7471441) |
| NN-BB1612-I | ERX4858714 | ERR5052599 | ERS5229146 (SAMEA7471450) |
| NN-BB456a-I | ERX4858715 | ERR5052600 | ERS5229148 (SAMEA7471452) |
| NW-BB1067-I | ERX4858716 | ERR5052601 | ERS5229139 (SAMEA7471443) |
| NW-BB117-I | ERX4858717 | ERR5052602 | ERS5229120 (SAMEA7471424) |
| NW-BB143-I | ERX4858718 | ERR5052603 | ERS5229140 (SAMEA7471444) |
| NW-BB148-I | ERX4858719 | ERR5052604 | ERS5229138 (SAMEA7471442) |
| PP-BB6767-I | ERX4858720 | ERR5052605 | ERS5229131 (SAMEA7471435) |
| PP-BB6788-I | ERX4858721 | ERR5052606 | ERS5229121 (SAMEA7471425) |
| PP-BB6821-I | ERX4858722 | ERR5052607 | ERS5229122 (SAMEA7471426) |
| PP-BB6903-I | ERX4858723 | ERR5052608 | ERS5229127 (SAMEA7471431) |
| PP-BB6945-I1 | ERX4858724 | ERR5052609 | ERS5229123 (SAMEA7471427) |
| PP-BB6988-I | ERX4858725 | ERR5052610 | ERS5229124 (SAMEA7471428) |
| PP-BB7107-I1 | ERX4858726 | ERR5052611 | ERS5229132 (SAMEA7471436) |
| PP-BB7109-I | ERX4858727 | ERR5052612 | ERS5229128 (SAMEA7471432) |
| PP-BB7266-I | ERX4858728 | ERR5052613 | ERS5229125 (SAMEA7471429) |
| PP-BB7295-I | ERX4858729 | ERR5052614 | ERS5229129 (SAMEA7471433) |
| PP-BB7468-I | ERX4858730 | ERR5052615 | ERS5229130 (SAMEA7471434) |
| PP-BB8159-I | ERX4858731 | ERR5052616 | ERS5229126 (SAMEA7471430) |

Additional file 1: Table S4 – Metadata for (a) *S. haemolyticus* and (b) *S. epidermidis* NCBI genomes

1. *S. haemolyticus*

| NCBI | ST | Date | Country | Source | Clinical |
| --- | --- | --- | --- | --- | --- |
| ASM76406v1 | 1 | 2013 | Malaysia | human, sputum | Yes |
| 7068_7_10 | 1 | 2007 | Germany | blood, sepsis | Yes |
| 7068_7_6 | 1 | 2008 | Germany | blood, sepsis | Yes |
| 7068_7_5 | 1 | 2008 | Germany | blood, sepsis | Yes |
| 7067_4_78 | 1 | 2010 | Belgium | horse | No |
| 7068_7_48 | 1 | 2004 | Switzerland | human | Yes |
| 7068_7_43 | 1 | 2005 | Switzerland | catheter | Yes |
| 7068_7_63 | 1 | 2006 | Switzerland | catheter | Yes |
| 7068_7_9 | 1 | 2008 | Germany | blood, sepsis | Yes |
| 7068_7_1 | 1 | 2005 | UK | human | Yes |
| 7068_7_49 | 1 | 2006 | Switzerland | human | Yes |
| 7067_4_40 | 1 | 2005 | Norway | blood, sepsis | Yes |
| 7068_7_61 | 1 | 2008 | Switzerland | catheter | Yes |
| 7068_7_12 | 1 | 2007 | Spain | blood, sepsis | Yes |
| 7068_7_51 | 1 | 2008 | Switzerland | blood, sepsis | Yes |
| 7068_7_42 | 1 | 2003 | Switzerland | catheter | Yes |
| 7068_7_8 | 1 | 2008 | Germany | blood, sepsis | Yes |
| 7068_7_2 | 1 | 2005 | UK | human | Yes |
| 7068_7_53 | 1 | 2007 | Switzerland | human | Yes |
| 7068_7_41 | 1 | 2008 | Switzerland | catheter | Yes |
| 7068_7_54 | 1 | 2005 | Switzerland | catheter | Yes |
| 7068_7_67 | 1 | 2007 | Switzerland | blood, sepsis | Yes |
| 7068_7_39 | 1 | 2005 | Switzerland | catheter | Yes |
| 7067_4_83 | 1 | 2005 | UK | human | Yes |
| 7068_7_7 | 1 | 2008 | Germany | blood, sepsis | Yes |
| 7068_7_4 | 1 | 2008 | Germany | blood, sepsis | Yes |
| 7068_7_46 | 1 | 2008 | Switzerland | blood, sepsis | Yes |
| 7067_4_36 | 1 | 2005 | Norway | blood, sepsis | Yes |
| 7068_7_11 | 1 | 2007 | Germany | blood, sepsis | Yes |
| 7067_4_77 | 1 | 2010 | Belgium | animal, disease | No |
| 7067_4_53 | 1 | 2004 | Norway | blood, sepsis | Yes |
| 7067_4_41 | 1 | 2008 | Switzerland | blood, sepsis | Yes |
| 7068_7_56 | 1 | 2008 | Switzerland | blood, sepsis | Yes |
| 7068_7_3 | 1 | 2008 | Germany | blood, sepsis | Yes |
| ASM152942v1 | 1 | 2010 | India | keratitis, eye | Yes |
| ASM152944v1 | 1 | 2010 | India | keratitis, eye | Yes |
| ASM359632v1 | 1 | 2017 | India | blood, sepsis | Yes |
| ASM504755v1 | 1 | ND | ND | human | ND |
| ASM504840v1 | 1 | ND | ND | human | ND |
| ASM504848v1 | 1 | ND | ND | human | ND |
| ASM514626v2 | 1 | ND | ND | human | ND |
| ASM767755v1 | 1 | 2018 | USA | Environmental | No |
| ASM918918v2 | 1 | 2017 | India | blood, sepsis | Yes |
| ASM1477975v1 | 1 | 2009 | China | cerebrospinal fluid | Yes |
| ASM107187v1 | 3 | ND | USA | human | ND |
| ASM107209v1 | 3 | ND | USA | human | ND |
| 7067_4_88 | 3 | 2005 | UK | human | Yes |
| 7067_4_51 | 3 | 2002 | Norway | blood, sepsis | Yes |
| 7068_7_52 | 3 | 2002 | Switzerland | human | Yes |
| 7067_4_44 | 3 | 2004 | Norway | blood, sepsis | Yes |
| 7068_7_29 | 3 | 2001 | Switzerland | catheter | Yes |
| 7067_4_38 | 3 | 2004 | Norway | blood, sepsis | Yes |
| 7067_4_46 | 3 | 2001 | Norway | blood, sepsis | Yes |
| 7068_7_30 | 3 | 2008 | Switzerland | blood, sepsis | Yes |
| 7068_7_36 | 3 | 2006 | Switzerland | catheter | Yes |
| 7067_4_66 | 3 | 1994 | Norway | blood, sepsis | Yes |
| 7068_7_38 | 3 | 2004 | Switzerland | blood, sepsis | Yes |
| 7067_4_50 | 3 | 2001 | Norway | blood, sepsis | Yes |
| 7067_4_42 | 3 | 2004 | Norway | blood, sepsis | Yes |
| 7067_4_57 | 3 | 2005 | Norway | blood, sepsis | Yes |
| 7068_7_55 | 3 | 2002 | Switzerland | human | Yes |
| 7067_4_73 | 3 | 2010 | Norway | human, nose | ND |
| 7068_7_31 | 3 | 2002 | Switzerland | blood, sepsis | Yes |
| 7067_4_37 | 3 | 2001 | Norway | blood, sepsis | Yes |
| 7068_7_15 | 3 | 2014 | Japan | human | Yes |
| 7067_4_43 | 3 | 2004 | Norway | blood, sepsis | Yes |
| 7067_4_90 | 3 | 2001 | Norway | blood, sepsis | Yes |
| 7067_4_54 | 3 | 2003 | Norway | blood, sepsis | Yes |
| 7068_7_45 | 3 | 2002 | Switzerland | human | Yes |
| ASM147151v2 | 3 | 2014 | USA | blood, sepsis - 18months | Yes |
| ASM170515v1 | 3 | 2011 | Russia | hospital env | No |
| ASM170524v1 | 3 | 2011 | Russia | neonatal care unit, human | No |
| ASM174342v1 | 3 | 2015 | ND | International Space Station | No |
| ASM196987v1 | 3 | 2012 | China | animal milk | No |
| ASM216543v1 | 3 | 2013 | Iran | blood, sepsis | Yes |
| ASM279954v1 | 3 | 2017 | Brazil | blood, sepsis | Yes |
| ASM342854v1 | 3 | 2009 | Russia | blood, sepsis | Yes |
| ASM342855v1 | 3 | 2009 | Russia | blood, sepsis | Yes |
| ASM359636v3 | 3 | 2017 | India | blood, sepsis | Yes |
| ASM395600v1 | 3 | 2016 | ND | BAL, child, human | Yes |
| ASM514619v2 | 3 | ND | ND | Human | Yes |
| ASM766609v1 | 3 | 2018 | USA | Environmental | No |
| ASM766719v1 | 3 | 2018 | USA | Environmental | No |
| ASM766803v1 | 3 | 2018 | USA | Environmental | No |
| ASM767771v1 | 3 | 2018 | USA | Environmental | No |
| ASM1426579v1 | 3 | 2015 | USA | International space station | No |
| ASM1426692v1 | 3 | 2015 | USA | International space station | No |
| ASM1426694v1 | 3 | 2015 | USA | International space station | No |
| 57-05 | 3 | 2013 | Norway | human | No |
| 57-32 | 3 | 2013 | Norway | human | No |
| 57-35 | 3 | 2014 | Norway | human | No |
| 57-47 | 3 | 2014 | Norway | human | No |
| 58-39 | 3 | 2014 | Norway | human | No |
| ASM107155v1 | 30 | ND | USA | human | ND |
| 7068_7_64 | 30 | 2008 | Switzerland | catheter | Yes |
| ASM359638v1 | 30 | 2017 | India | blood, sepsis | Yes |
| ASM767958v1 | 30 | 2018 | USA | Environmental | No |
| ASM768035v1 | 30 | 2018 | USA | Environmental | No |
| ASM227697v1 | 56 | 2016 | Germany | Kefir | No |
| ASM27608v1 | 2 | 2004 | USA | blood, sepsis | Yes |
| ASM165924v1 | 2 | 2004 | Germany | ENV clinical | No |
| ASM27610v1 | 2 | 2005 | USA | blood, sepsis | Yes |
| ASM27612v1 | 2 | 2005 | USA | blood, sepsis | Yes |
| ASM285031v1 | 2 | 2007 | USA | blood, sepsis | Yes |
| ASM165922v1 | 2 | 2008 | Germany | ENV | No |
| ASM165930v1 | 2 | 2008 | Poland | ENV clinical | No |
| ASM303536v1 | 2 | 2008 | Canada | animal | No |
| ASM1017222v1 | 2 | 2010 | USA | skin | No |
| ASM1017251v1 | 2 | 2010 | USA | skin | No |
| ASM437479v1 | 2 | 2011 | Mexico | blood, sepsis, newborn | Yes |
| ASM892231v1 | 2 | 2011 | UK | blood, sepsis | Yes |
| Se_BPH0736 | 2 | 2011 | Australia | human | Yes |
| ASM902604v1 | 2 | 2012 | UK | blood, sepsis | Yes |
| ASM162287v1 | 2 | 2014 | Germany | human | No |
| ASM165888v1 | 2 | 2014 | Germany | human | No |
| ASM165889v1 | 2 | 2014 | Germany | human | No |
| ASM165890v1 | 2 | 2014 | Germany | human | No |
| ASM165894v1 | 2 | 2014 | Germany | human | No |
| ASM165898v1 | 2 | 2014 | Germany | human | No |
| ASM165906v1 | 2 | 2014 | Germany | human | No |
| ID412assemblyCLC | 2 | 2014 | Italy | knee joint infection, human | Yes |
| ASM275014v1 | 2 | 2015 | USA | human, neck | Yes |
| ASM368736v1 | 2 | 2016 | USA | human | ND |
| ASM892279v1 | 2 | 2017 | UK | blood, sepsis | Yes |
| ASM767721v1 | 2 | 2018 | USA | ENV | No |
| gsevcu014v01 | 2 | ND | ND | ND | ND |
| gsevcu013v01 | 2 | ND | ND | ND | ND |
| ASM106889v1 | 2 | ND | USA | ND | ND |
| ASM107101v1 | 2 | ND | USA | ND | ND |
| ASM902609v1 | 210 | 2012 | UK | blood, sepsis | Yes |
| ASM892275v1 | 210 | 2017 | UK | blood, sepsis | Yes |
| ASM892284v1 | 210 | 2017 | UK | blood, sepsis | Yes |
| ASM107271v1 | 210 | ND | USA | Human | ND |
| ASM107277v1 | 210 | ND | USA | Human | ND |
| ASM96671v1 | 439 | 2013 | UK | neonatal sepsis | Yes |
| 25426_7_61 | 439 | 2018 | UK | faecal carriage | No |
| ASM989700v1 | 490 | ND | Ireland | human | ND |

(b) *S. epidermidis*

Additional file 1: Table S5 – Metadata for the NCBI genomes incorporated into *S. scuiri* SNP analysis.

Additional file 1: Table S6 - The numbers of microbial isolates recovered from blood cultures from each clinical site in BARNARDS. The number of neonates with a Gram-positive or Gram-negative sepsis are listed per clinical site. The number of neonates reported as deceased is listed for each GPB or GNB per clinical site. Data based on neonates instead of number of isolates recorded from a blood culture also includes whether both a GPB and GNB was recovered from the blood culture. Comparison between GPB and GNB was performed using the two-sample test for equality of proportions with continuity correction.

|  |  |  | Africa |  |  |  |  |  |  | South Asia |  |  |  |  |  |
| --- | --- | --- | --- | --- | --- | --- | --- | --- | --- | --- | --- | --- | --- | --- | --- |
|  |  |  | Ethiopia | Nigeria |  |  | Rwanda |  | South Africa | Bangladesh |  | India | Pakistan |  | TOTAL |
|  |  |  | ES | NK | NN | NW | RK | RU | ZAT | BC | BK | IN | PC | PP |  |
| (A) Enrolment and prevalence data | Neonates enrolled |  | 4828 | 7319 | 1902 | 2359 | 2255 | 1222 | 3312 | 1772 | 1707 | 1160 | 504 | 7945 | 36285 |
|  | Neonates with reported culture confirmed |  | 443 | 315 | 228 | 62 | 240 | 65 | 94 | 192 | 19 | 21 | 37 | 767 | 2483 |
|  | Neonates with a confirmed **bacterial** blood culture |  | 285 | 258 | 220 | 54 | 219 | 57 | 85 | 192 | 19 | 17 | 41 | 785 | 2232 |
|  | Isolates recovered from blood cultures | GNB | 150 | 77 | 92 | 7 | 58 | 22 | 39 | 170 | 14 | 14 | 20 | 375 | 1038 |
|  |  | GPB | 141 | 183 | 137 | 48 | 161 | 35 | 73 | 25 | 5 | 3 | 28 | 427 | 1266 |
|  |  | Fungi | 9 | 3 | 5 | 0 | 0 | 0 | 0 | 0 | 0 | 3 | 0 | 2 | 22 |
|  |  | NA | 30 | 0 | 0 | 0 | 12 | 4 | 1 | 0 | 0 | 0 | 0 | 1 | 48 |
|  |  | Total | 330 | 263 | 234 | 55 | 231 | 61 | 113 | 195 | 19 | 20 | 48 | 805 | 2374 |
| (B) Comparison of the incidence of GNB and GPB sepsis | Neonates with a positive blood culture | GNB | 147 | 76 | 89 | 7 | 58 | 22 | 32 | 170 | 14 | 14 | 15 | 363 | 1007 |
|  |  | GPB | 138 | 182 | 131 | 47 | 161 | 35 | 64 | 22 | 5 | 3 | 26 | 422 | 1236 |
|  |  | GNB and GPB | 5 | 1 | 1 | 1 | 0 | 0 | 11 | 0 | 0 | 0 | 6 | 21 | 46 |
|  |  | Sig(p) | 0.6305 | <0.0001 | 0.0044 | <0.0001 | <0.0001 | 0.1078 | 0.0014 | 0.0000 | 0.0657 | 0.0149 | 0.1108 | 0.0337 | <0.0001 |
| (C) Comparison of mortality following biological sepsis (MfBS) of GNB and GPB sepsis | Mortality following biological sepsis (MfBS) | GNB | 23 | 19 | 30 | 0 | 3 | 4 | 7 | 13 | 1 | 4 | 7 | 86 | 197 |
|  |  | GPB | 26 | 10 | 14 | 4 | 3 | 1 | 13 | 0 | 0 | 0 | 6 | 43 | 120 |
|  |  | GNB and GPB | 0 | 0 | 0 | 0 | - | - | 3 | - | - | - | 3 | 7 | 13 |

Additional file 1: Table S7 – VF gene presence/absence data. VF hits from the WGS data were analysed according to the presence of the gene against two clinical datasets, the onset of sepsis (EOS v LOS) and the outcome (alive v deceased). Significance was taken at p=<0.05.

|  | **Onset** | | | | | | **Outcome** | | | | | |
| --- | --- | --- | --- | --- | --- | --- | --- | --- | --- | --- | --- | --- |
| **Gene** | **EOS absent** | **EOS present** | **LOS absent** | **LOS present** | **Pvalue** | **Adj_Pvalue** | **Alive absent** | **Alive present** | **Deceased absent** | **Deceased alive** | **Pvalue** | **Adjusted_Pvalue** |
| aur | 8 (20%) | 32 (80%) | 6 (13%) | 40 (87%) | 0.383 | 0.600 | 18 (22%) | 64 (78%) | 0 (0%) | 18 (100%) | 0.028 | 0.335 |
| bsh | 40 (100%) | 0 (0%) | 45 (97.8%) | 1 (2.2%) | 0.348 | 0.582 | 81 (98.8%) | 1 (1.2%) | 18 (100%) | 0 (0%) | 0.638 | 0.778 |
| cap8A | 4 (10%) | 36 (90%) | 11 (23.9%) | 35 (76.1%) | 0.090 | 0.379 | 13 (15.9%) | 69 (84.1%) | 2 (11.1%) | 16 (88.9%) | 0.610 | 0.778 |
| cap8B | 12 (30%) | 28 (70%) | 17 (37%) | 29 (63%) | 0.496 | 0.696 | 31 (37.8%) | 51 (62.2%) | 2 (11.1%) | 16 (88.9%) | 0.029 | 0.335 |
| cap8C | 1 (2.5%) | 39 (97.5%) | 0 (0%) | 46 (100%) | 0.281 | 0.535 | 1 (1.2%) | 81 (98.8%) | 0 (0%) | 18 (100%) | 0.638 | 0.778 |
| cap8H | 22 (55%) | 18 (45%) | 37 (80.4%) | 9 (19.6%) | 0.011 | 0.269 | 50 (61%) | 32 (39%) | 13 (72.2%) | 5 (27.8%) | 0.371 | 0.694 |
| cap8I | 22 (55%) | 18 (45%) | 36 (78.3%) | 10 (21.7%) | 0.022 | 0.269 | 48 (58.5%) | 34 (41.5%) | 14 (77.8%) | 4 (22.2%) | 0.128 | 0.504 |
| cap8J | 23 (57.5%) | 17 (42.5%) | 35 (76.1%) | 11 (23.9%) | 0.067 | 0.379 | 49 (59.8%) | 33 (40.2%) | 13 (72.2%) | 5 (27.8%) | 0.324 | 0.689 |
| cap8K | 21 (52.5%) | 19 (47.5%) | 35 (76.1%) | 11 (23.9%) | 0.022 | 0.269 | 47 (57.3%) | 35 (42.7%) | 13 (72.2%) | 5 (27.8%) | 0.242 | 0.677 |
| chp | 24 (60%) | 16 (40%) | 32 (69.6%) | 14 (30.4%) | 0.353 | 0.582 | 53 (64.6%) | 29 (35.4%) | 11 (61.1%) | 7 (38.9%) | 0.778 | 0.877 |
| clfA | 30 (75%) | 10 (25%) | 41 (89.1%) | 5 (10.9%) | 0.085 | 0.379 | 71 (86.6%) | 11 (13.4%) | 13 (72.2%) | 5 (27.8%) | 0.132 | 0.504 |
| clfB | 39 (97.5%) | 1 (2.5%) | 44 (95.7%) | 2 (4.3%) | 0.641 | 0.815 | 78 (95.1%) | 4 (4.9%) | 18 (100%) | 0 (0%) | 0.339 | 0.689 |
| cna | 31 (77.5%) | 9 (22.5%) | 38 (82.6%) | 8 (17.4%) | 0.553 | 0.750 | 61 (74.4%) | 21 (25.6%) | 14 (77.8%) | 4 (22.2%) | 0.764 | 0.877 |
| coa | 37 (92.5%) | 3 (7.5%) | 43 (93.5%) | 3 (6.5%) | 0.859 | 0.901 | 76 (92.7%) | 6 (7.3%) | 16 (88.9%) | 2 (11.1%) | 0.591 | 0.778 |
| ebp | 2 (5%) | 38 (95%) | 4 (8.7%) | 42 (91.3%) | 0.502 | 0.696 | 5 (6.1%) | 77 (93.9%) | 1 (5.6%) | 17 (94.4%) | 0.930 | 0.946 |
| esaA | 1 (2.5%) | 39 (97.5%) | 0 (0%) | 46 (100%) | 0.281 | 0.535 | 1 (1.2%) | 81 (98.8%) | 0 (0%) | 18 (100%) | 0.638 | 0.778 |
| esaB | 2 (5%) | 38 (95%) | 6 (13%) | 40 (87%) | 0.200 | 0.452 | 10 (12.2%) | 72 (87.8%) | 0 (0%) | 18 (100%) | 0.118 | 0.504 |
| esaD | 11 (27.5%) | 29 (72.5%) | 7 (15.2%) | 39 (84.8%) | 0.163 | 0.417 | 22 (26.8%) | 60 (73.2%) | 1 (5.6%) | 17 (94.4%) | 0.052 | 0.353 |
| esaE | 6 (15%) | 34 (85%) | 6 (13%) | 40 (87%) | 0.794 | 0.901 | 16 (19.5%) | 66 (80.5%) | 0 (0%) | 18 (100%) | 0.041 | 0.335 |
| esaG1 | 29 (72.5%) | 11 (27.5%) | 40 (87%) | 6 (13%) | 0.093 | 0.379 | 61 (74.4%) | 21 (25.6%) | 15 (83.3%) | 3 (16.7%) | 0.421 | 0.694 |
| esaG2 | 30 (75%) | 10 (25%) | 40 (87%) | 6 (13%) | 0.155 | 0.417 | 62 (75.6%) | 20 (24.4%) | 15 (83.3%) | 3 (16.7%) | 0.481 | 0.728 |
| esaG3 | 31 (77.5%) | 9 (22.5%) | 40 (87%) | 6 (13%) | 0.249 | 0.506 | 65 (79.3%) | 17 (20.7%) | 15 (83.3%) | 3 (16.7%) | 0.696 | 0.817 |
| esaG4 | 30 (75%) | 10 (25%) | 40 (87%) | 6 (13%) | 0.155 | 0.417 | 62 (75.6%) | 20 (24.4%) | 15 (83.3%) | 3 (16.7%) | 0.481 | 0.728 |
| esaG5 | 26 (65%) | 14 (35%) | 26 (56.5%) | 20 (43.5%) | 0.423 | 0.644 | 50 (61%) | 32 (39%) | 8 (44.4%) | 10 (55.6%) | 0.198 | 0.672 |
| esaG6 | 38 (95%) | 2 (5%) | 45 (97.8%) | 1 (2.2%) | 0.476 | 0.692 | 77 (93.9%) | 5 (6.1%) | 18 (100%) | 0 (0%) | 0.282 | 0.677 |
| esaG7 | 38 (95%) | 2 (5%) | 46 (100%) | 0 (0%) | 0.125 | 0.385 | 77 (93.9%) | 5 (6.1%) | 18 (100%) | 0 (0%) | 0.282 | 0.677 |
| esaG8 | 39 (97.5%) | 1 (2.5%) | 45 (97.8%) | 1 (2.2%) | 0.920 | 0.920 | 77 (93.9%) | 5 (6.1%) | 18 (100%) | 0 (0%) | 0.282 | 0.677 |
| esaG9 | 37 (92.5%) | 3 (7.5%) | 43 (93.5%) | 3 (6.5%) | 0.859 | 0.901 | 78 (95.1%) | 4 (4.9%) | 15 (83.3%) | 3 (16.7%) | 0.076 | 0.433 |
| essC | 6 (15%) | 34 (85%) | 6 (13%) | 40 (87%) | 0.794 | 0.901 | 16 (19.5%) | 66 (80.5%) | 0 (0%) | 18 (100%) | 0.041 | 0.335 |
| fnbA | 35 (87.5%) | 5 (12.5%) | 39 (84.8%) | 7 (15.2%) | 0.717 | 0.874 | 70 (85.4%) | 12 (14.6%) | 17 (94.4%) | 1 (5.6%) | 0.300 | 0.677 |
| fnbB | 38 (95%) | 2 (5%) | 44 (95.7%) | 2 (4.3%) | 0.886 | 0.901 | 76 (92.7%) | 6 (7.3%) | 17 (94.4%) | 1 (5.6%) | 0.791 | 0.877 |
| geh | 8 (20%) | 32 (80%) | 22 (47.8%) | 24 (52.2%) | 0.007 | 0.269 | 25 (30.5%) | 57 (69.5%) | 7 (38.9%) | 11 (61.1%) | 0.489 | 0.728 |
| hlb | 36 (90%) | 4 (10%) | 34 (73.9%) | 12 (26.1%) | 0.056 | 0.379 | 68 (82.9%) | 14 (17.1%) | 16 (88.9%) | 2 (11.1%) | 0.532 | 0.773 |
| hlgB | 11 (27.5%) | 29 (72.5%) | 17 (37%) | 29 (63%) | 0.351 | 0.582 | 29 (35.4%) | 53 (64.6%) | 2 (11.1%) | 16 (88.9%) | 0.044 | 0.335 |
| hlgC | 8 (20%) | 32 (80%) | 17 (37%) | 29 (63%) | 0.084 | 0.379 | 26 (31.7%) | 56 (68.3%) | 2 (11.1%) | 16 (88.9%) | 0.078 | 0.433 |
| hly.hla | 9 (22.5%) | 31 (77.5%) | 11 (23.9%) | 35 (76.1%) | 0.877 | 0.901 | 19 (23.2%) | 63 (76.8%) | 2 (11.1%) | 16 (88.9%) | 0.255 | 0.677 |
| hysA | 39 (97.5%) | 1 (2.5%) | 44 (95.7%) | 2 (4.3%) | 0.641 | 0.815 | 78 (95.1%) | 4 (4.9%) | 18 (100%) | 0 (0%) | 0.339 | 0.689 |
| icaB | 3 (7.5%) | 37 (92.5%) | 3 (6.5%) | 43 (93.5%) | 0.859 | 0.901 | 5 (6.1%) | 77 (93.9%) | 2 (11.1%) | 16 (88.9%) | 0.450 | 0.723 |
| icaC | 1 (2.5%) | 39 (97.5%) | 4 (8.7%) | 42 (91.3%) | 0.221 | 0.481 | 5 (6.1%) | 77 (93.9%) | 0 (0%) | 18 (100%) | 0.282 | 0.677 |
| isdA | 9 (22.5%) | 31 (77.5%) | 11 (23.9%) | 35 (76.1%) | 0.877 | 0.901 | 20 (24.4%) | 62 (75.6%) | 2 (11.1%) | 16 (88.9%) | 0.218 | 0.677 |
| isdB | 4 (10%) | 36 (90%) | 11 (23.9%) | 35 (76.1%) | 0.090 | 0.379 | 13 (15.9%) | 69 (84.1%) | 2 (11.1%) | 16 (88.9%) | 0.610 | 0.778 |
| lukD | 13 (32.5%) | 27 (67.5%) | 17 (37%) | 29 (63%) | 0.665 | 0.828 | 32 (39%) | 50 (61%) | 2 (11.1%) | 16 (88.9%) | 0.024 | 0.335 |
| map | 33 (82.5%) | 7 (17.5%) | 43 (93.5%) | 3 (6.5%) | 0.113 | 0.384 | 75 (91.5%) | 7 (8.5%) | 15 (83.3%) | 3 (16.7%) | 0.298 | 0.677 |
| PVL | 30 (75%) | 10 (25%) | 27 (58.7%) | 19 (41.3%) | 0.111 | 0.384 | 59 (72%) | 23 (28%) | 11 (61.1%) | 7 (38.9%) | 0.363 | 0.694 |
| sak | 1 (2.5%) | 39 (97.5%) | 9 (19.6%) | 37 (80.4%) | 0.014 | 0.269 | 7 (8.5%) | 75 (91.5%) | 4 (22.2%) | 14 (77.8%) | 0.093 | 0.472 |
| sbi | 14 (35%) | 26 (65%) | 21 (45.7%) | 25 (54.3%) | 0.316 | 0.567 | 37 (45.1%) | 45 (54.9%) | 3 (16.7%) | 15 (83.3%) | 0.026 | 0.335 |
| scn | 1 (2.5%) | 39 (97.5%) | 7 (15.2%) | 39 (84.8%) | 0.043 | 0.379 | 5 (6.1%) | 77 (93.9%) | 4 (22.2%) | 14 (77.8%) | 0.030 | 0.335 |
| sdrC | 37 (92.5%) | 3 (7.5%) | 45 (97.8%) | 1 (2.2%) | 0.242 | 0.506 | 78 (95.1%) | 4 (4.9%) | 17 (94.4%) | 1 (5.6%) | 0.905 | 0.936 |
| sdrE | 15 (37.5%) | 25 (62.5%) | 21 (45.7%) | 25 (54.3%) | 0.445 | 0.662 | 39 (47.6%) | 43 (52.4%) | 5 (27.8%) | 13 (72.2%) | 0.126 | 0.504 |
| sea | 27 (67.5%) | 13 (32.5%) | 39 (84.8%) | 7 (15.2%) | 0.058 | 0.379 | 58 (70.7%) | 24 (29.3%) | 14 (77.8%) | 4 (22.2%) | 0.547 | 0.775 |
| seb | 34 (85%) | 6 (15%) | 43 (93.5%) | 3 (6.5%) | 0.200 | 0.452 | 74 (90.2%) | 8 (9.8%) | 15 (83.3%) | 3 (16.7%) | 0.396 | 0.694 |
| sec | 37 (92.5%) | 3 (7.5%) | 38 (82.6%) | 8 (17.4%) | 0.171 | 0.417 | 72 (87.8%) | 10 (12.2%) | 17 (94.4%) | 1 (5.6%) | 0.415 | 0.694 |
| seh | 38 (95%) | 2 (5%) | 44 (95.7%) | 2 (4.3%) | 0.886 | 0.901 | 78 (95.1%) | 4 (4.9%) | 17 (94.4%) | 1 (5.6%) | 0.905 | 0.936 |
| selk | 37 (92.5%) | 3 (7.5%) | 42 (91.3%) | 4 (8.7%) | 0.840 | 0.901 | 76 (92.7%) | 6 (7.3%) | 16 (88.9%) | 2 (11.1%) | 0.591 | 0.778 |
| sell | 37 (92.5%) | 3 (7.5%) | 38 (82.6%) | 8 (17.4%) | 0.171 | 0.417 | 72 (87.8%) | 10 (12.2%) | 17 (94.4%) | 1 (5.6%) | 0.415 | 0.694 |
| selq | 35 (87.5%) | 5 (12.5%) | 42 (91.3%) | 4 (8.7%) | 0.565 | 0.750 | 74 (90.2%) | 8 (9.8%) | 15 (83.3%) | 3 (16.7%) | 0.396 | 0.694 |
| spa | 19 (47.5%) | 21 (52.5%) | 27 (58.7%) | 19 (41.3%) | 0.299 | 0.553 | 43 (52.4%) | 39 (47.6%) | 9 (50%) | 9 (50%) | 0.851 | 0.927 |
| srtB | 1 (2.5%) | 39 (97.5%) | 3 (6.5%) | 43 (93.5%) | 0.377 | 0.600 | 4 (4.9%) | 78 (95.1%) | 1 (5.6%) | 17 (94.4%) | 0.905 | 0.936 |
| sspA | 16 (40%) | 24 (60%) | 26 (56.5%) | 20 (43.5%) | 0.126 | 0.385 | 41 (50%) | 41 (50%) | 8 (44.4%) | 10 (55.6%) | 0.669 | 0.801 |
| tsst.1 | 33 (82.5%) | 7 (17.5%) | 43 (93.5%) | 3 (6.5%) | 0.113 | 0.384 | 73 (89%) | 9 (11%) | 16 (88.9%) | 2 (11.1%) | 0.987 | 0.987 |
| vWbp | 30 (75%) | 10 (25%) | 41 (89.1%) | 5 (10.9%) | 0.085 | 0.379 | 70 (85.4%) | 12 (14.6%) | 13 (72.2%) | 5 (27.8%) | 0.179 | 0.642 |

Additional file 1: Figure S1 – Bioinformatics workflow for this study, detailing the different approaches and programs used.


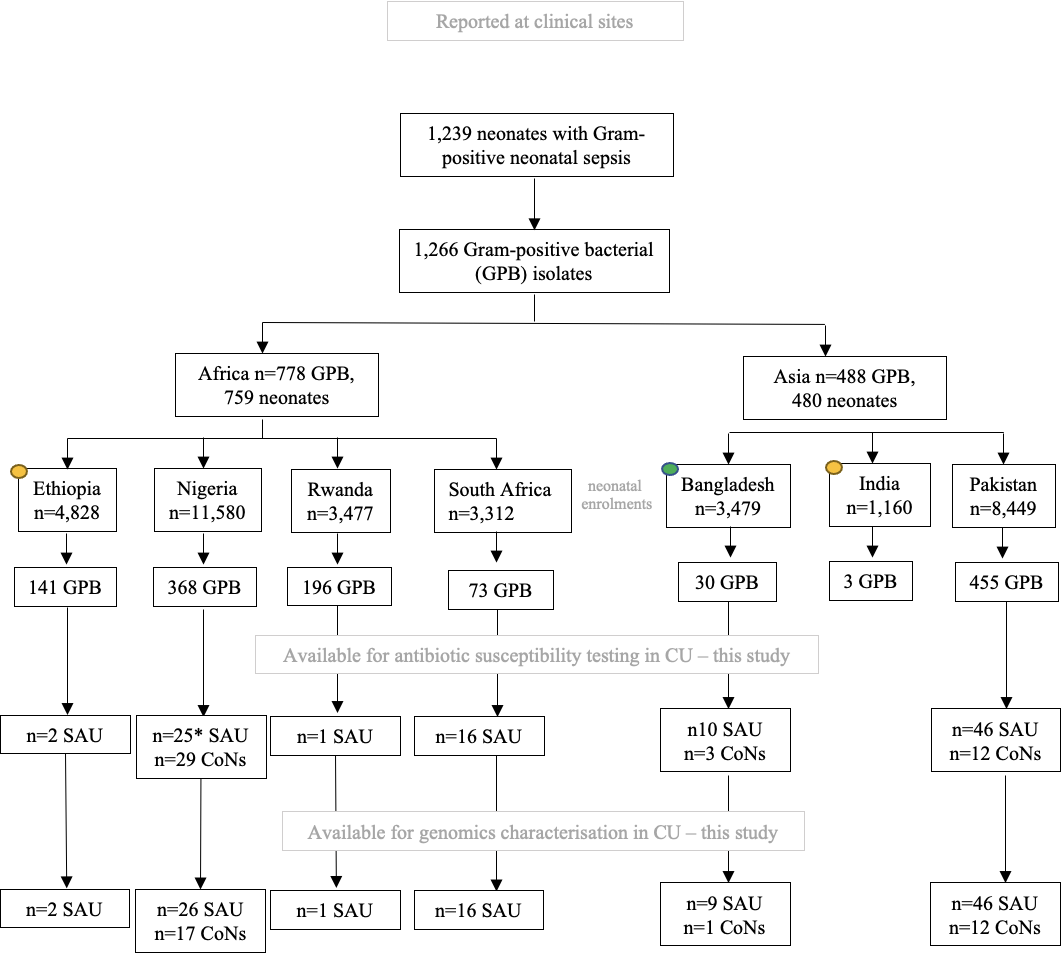


Additional file 1: Figure S2 – A flow chart to describe the enrolment data, the incidence of Gram-positive sepsis, the total number of Gram-positive bacterial isolates recovered from blood cultures, the number per country, how many were available for antibiotic susceptibility testing, and how many were available for WGS analysis. Not all clinical sites had the capacity to keep GPB isolates during BARNARDS, and only certain sites and/or species (following local identification) were kept and available for transportation. Green indicates sites that did not routinely collect and report GPB and yellow indicates sites with a later enrolment start date.

Additional file 1: Figure S3 - A stacked bar graph listing the 14 different GPB species identified using either MALDI-TOF MS and for Staphylococci was confirmed with WGS. A total of 970 isolates were recorded as GPB however identification and appropriate storage of bacterial isolates was not performed. This data is not represented in the graph. SAU – *S. aures,* SEP – *S. epidermidis,* SHE – *S. haemolyicus,* SSC – *M. sciuri,* EFL – *Enterococcus faecalis,* EFC – *Enterococcus faecium,* BCR – ACV – *Aerococcus viridans,* ECA – EGL – BRC – *Brevibacterium casei,* DNT – *Dietzia natronolimnaea*, EXA – *Exiguobacterium aurantiacum,* EXM - *Exiguobacterium mexicanum*

Additional file 1: Figure S4 - A stacked bar graph showing the onset of sepsis (EOS – early onset, LOS – late onset, ND – onset not determined) for each Staphylococci species.

SAU – *S. aureus,* SEP – *S. epidermidis,* SHE – *S. haemolyticus,* MSC - *Mammaliicoccus sciuri*

Additional file 1: Figure S5. A core genome SNP tree of ST5 *S. aureus* identified within the study, generated using IQ-tree. ASM2458v1 was selected as the reference from the complete genome assemblies within NCBI.

Additional file 1: Figure S6. A core genome SNP tree of ST152 *S. aureus* identified within the study, generated using IQ-tree. ASM1103510v1 was selected as the reference from the complete genome assemblies within NCBI.

Additional file 1: Figure S7. A core genome SNP tree of ST6 *S. aureus* identified within the study, generated using IQ-tree. ASM1103510v1 was selected as the reference from the complete genome assemblies within NCBI.

Additional file 1: Figure S8. A core genome SNP tree of ST8 *S. aureus* identified within the study, generated using IQ-tree. ASM118372V2 was selected as the reference from the complete genome assemblies within NCBI.

Additional file 1: Figure S9. A core genome SNP tree of ST30 *S. haemolyticus* identified within the study and those found from the NCBI database. The country the isolate was found in is demarcated by the branch symbol colour (Green = Switzerland, red = USA, Orange = India. The shades of blue represent the different clinical sites within Nigeria from this study). The source of isolate is abbreviated at the end of the branches (ENV = environmental, NEO-SEP = neonatal sepsis, CLIN = human clinical, HU = human, SEP = sepsis). All other available metadata can be found in Additional file 1: Table 2.

Additional file 1: Figure S10. A core genome SNP tree of ST1 *S. haemolyticus* identified within the study and those found from the NCBI database. The country the isolate was found in is demarcated by the branch symbol colour and accompanying key. The source of isolate is abbreviated at the end of the branches (ANI = animal, ENV = environmental, NEO-SEP = neonatal sepsis, CLIN = human clinical, HU = human, SEP = sepsis). All other available metadata can be found in Additional file 1: Table 2.

Additional file 1: Figure S11. A core genome SNP tree of ST3 *S. haemolyticus* identified within the study and those found from the NCBI database. The country the isolate was found in is demarcated by the branch symbol colour and accompanying key. The source of isolate is abbreviated at the end of the branches (ANI = animal, ENV = environmental, NEO-SEP = neonatal sepsis, CLIN = human clinical, HU = human, SEP = sepsis, OTH = other). All other available metadata can be found in Additional file 1: Table 2.

Additional file 1: Figure S12. A core genome SNP tree of ST2 *S. epidermidis* identified within the study and those found from the NCBI database. The country the isolate was found in is demarcated by the branch symbol colour and accompanying key and was focused primarily to the clade in which the isolate within this study was found (denoted by a larger circle). The source of isolate is abbreviated at the end of the branches (ANI = animal, ENV = environmental, NEO-SEP = neonatal sepsis, CLIN = human clinical, HU = human, SEP = sepsis, ND = not determined). All available metadata can be found in Additional file 1: Table 2.

Additional file 1: Figure S13 – A heatmap of VF-1 score (VFs previously suggested to be associated with *S. aureus* sepsis) generated using the *pheatmap* package in R 3.6.2


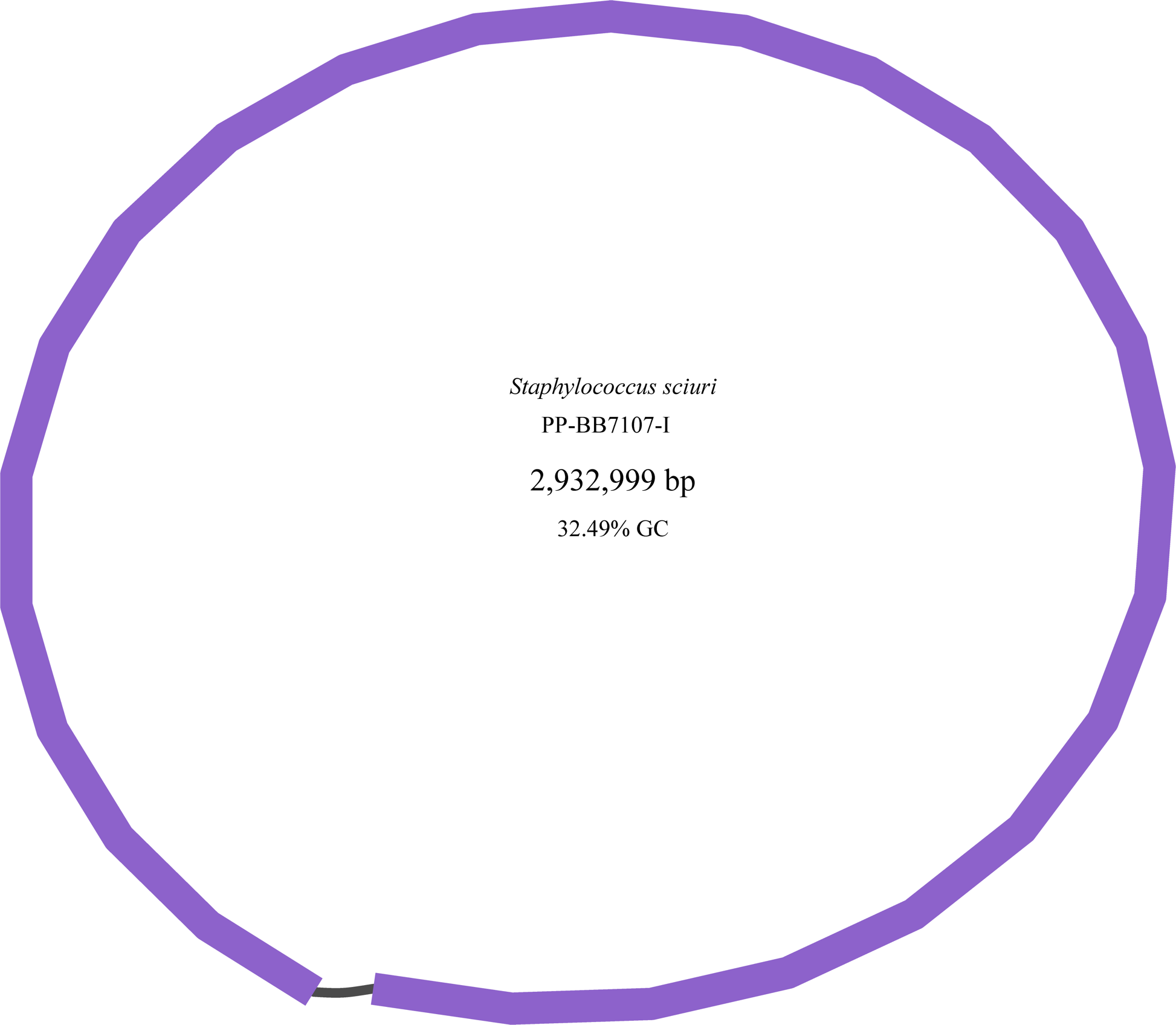


Additional file 1: Figure S14 – Chromosome of *S. scuiri* used as the reference genome for SNP analysis visualised using Bandage (v0.8.1) with the assembly graph of the Unicycler (v0.4.7) assembly with short reads generated during Illumina MiSeq sequencing. Genome accession number: CP071138 and biosample: SAMN17906023.
